# A Novel Multiattribute Decision-Making Method Based on Point–Choquet Aggregation Operators and Its Application in Supporting the Hierarchical Medical Treatment System in China

**DOI:** 10.3390/ijerph15081718

**Published:** 2018-08-10

**Authors:** Runtong Zhang, Yuping Xing, Jun Wang, Xiaopu Shang, Xiaomin Zhu

**Affiliations:** 1 School of Economics and Management, Beijing Jiaotong University, Beijing 100044, China; rtzhang@bjtu.edu.cn (R.Z.); 16113126@bjtu.edu.cn (Y.X.); 13120634@bjtu.edu.cn (J.W.); 2 School of Mechanical, Electronic and Control Engineering, Beijing Jiaotong University, Beijing 100044, China

**Keywords:** multiattribute decision making, picture fuzzy set, picture fuzzy point–Choquet aggregation operators, hierarchical medical treatment system

## Abstract

The hierarchical medical treatment system is an efficient way to solve the problem of insufficient and unbalanced medical resources in China. Essentially, classifying the different degrees of diseases according to the doctor’s diagnosis is a key step in pushing forward the hierarchical medical treatment system. This paper proposes a framework to solve the problem where diagnosis values are given as picture fuzzy numbers (PFNs). Point operators can reduce the uncertainty of doctor’s diagnosis and get intensive information in the process of decision making, and the Choquet integral operator can consider correlations among symptoms. In order to take full advantage of these two kinds of operators, in this paper, we firstly define some point operators under the picture fuzzy environment, and further propose a new class of picture fuzzy point–Choquet integral aggregation operators. Moreover, some desirable properties of these operators are also investigated in detail. Then, a novel approach based on these operators for multiattribute decision-making problems in the picture fuzzy context is introduced. Finally, we give an example to illustrate the applicability of the new approach in assisting hierarchical medical treatment system. This is of great significance for integrating the medical resources of the whole society and improving the service efficiency of the medical service system.

## 1. Introduction

With increasing environmental issues, lung diseases are becoming a serious health problem in China. As the medical facilities in grade III, class A hospitals are much better than those of in other small hospitals, people prefer to go to those relatively high-level hospitals for treatment. As a result, overcrowding in large hospitals is common, far exceeding the coping capacity. At the same time, however, small hospitals or clinics waste medical resources. Under such circumstances, how to better allocate limited medical resources and improve the input and output efficiency of the health care system are new challenges for the medical system in China.

Developing a hierarchical medical treatment system is regarded as key an effective way to solve the problem of insufficient and unbalanced medical resources, in which medical institutions at various levels receive patients according to the degree and urgency of the diseases they have. In such a system, common illnesses are treated at basic clinics, with patients transferred to more specialized facilities if their condition demands it. Serious illnesses should be treated in higher-grade hospitals. At the same time, higher-grade hospitals can also transfer patients down to lower-grade ones as their condition stabilizes. Thus, determining the severity of the illness is a key action in this system. At present, with the increase in the number of patients with lung diseases, establishing an appropriate approach to divide patients under different conditions into different levels of hospitals is an effective way to make full use of limited medical resources and cure more patients with lung diseases. However, the diagnosis for patient’s condition often involves multiple correlative criteria and thus can be described as multiattribute decision making (MADM) problems. This paper proposes a general framework in order to solve the MADM problem, which can be applied in the above scenario.

The essence of MADM is the process of ranking the alternatives and selecting an optimal scheme among a set of alternatives with respect to a list of attribute value. Recently, MADM has received much attention from scholars and has been widely applied to economic management and daily life. For example, Tang et al. [1] proposed an algorithm for group decision making with incomplete hesitant fuzzy linguistic preference relations and applied it to flood disaster risk evaluation. Qi [2] developed two effective multicriteria decision making (MCDM) approaches based on defined prioritized average aggregation operators and applied them to tackle complex emergency response solutions evaluation problems. Lin [3] proposed a linear program and a procedure for solving linguistic MADM problems with risk preferences and incomplete weight information, and further applied it to low-carbon tourism destination selection. Due to the increased complexity of real decision-making problems, we usually have to face the difficulty of representing attribute values appropriately. Chatterjee et al. [4] proposed a novel hybrid method encompassing factor relationship and multi-attributive border approximation area comparison methods for selection and evaluation of non-traditional machining process. Roya et al. [5] proposed a rough group analytic hierarchy process approach to the evaluation supplier criteria in the company for producing metal washers for the automotive industry. Vasiljević et al. [6] developed rough strength relational decision making and trial evaluation laboratory model to analyze the individual priorities of key success factors of hospital’s performance measures. As a generalization of the intuitionistic fuzzy set (IFS) [7], the picture fuzzy set (PFS) introduced by Cuong [8] is a very effective tool to express the complex fuzzy information because it is characterized by three functions expressing the degree of positive, neutral, and negative memberships at the same time. Because of this advantage, the PFS has been widely investigated and quite a few achievements have been made [9,10,11,12,13]. Among them, an important research topic in the research fields of MADM is aggregation operator theory, that can aggregate a collection of individual evaluated values into one. Abbas et al. [14] presented a comprehensive review on aggregation operator theory and decision-making approaches between 1986 and 2017. Among these aggregation operators, traditional aggregation operators, such as arithmetic and geometric operators for the IFS and neutral averaging operators [15] are based on the assumption that the attributes are independent of one another. However, the attributes of the problem are often correlative in the real decision-making process, especially in medical diagnosis. For example, to evaluate patients based on the following symptoms of lung diseases: (vital signs, body temperature, cough and hemoptysis), we want to place more emphasis on hemoptysis than on body temperature. However, on the other hand, we also want to pay more attention to patients who have severe hemoptysis and high body temperature, because hemoptysis and hyperthermia are two classical symptoms of pneumonia. Therefore, we need to find some new ways to deal with these situations where the decision data are correlative. The Choquet integral [16] introduced by Choquet is a useful tool to address the problem. Many scholars have made quite a few achievements in this field and applied the Choquet integral in MADM problems. By using Choquet integral and quasi-arithmetic means, Zhou and Chen [17] proposed a combined continuous quasi-arithmetic Choquet integral operator and a combined continuous generalized Choquet integral operator. In order to globally reflect the interactions between elements, Meng and Zhang [18] further defined the probabilistic generalized semivalue-induced continuous Choquet weighted averaging operator and the induced continuous Choquet geometric mean operator. Xu [19] used the Choquet integral to propose some operators for aggregating intuitionistic fuzzy values with correlative weights and further extended those operators to interval-valued intuitionistic fuzzy sets. Yager [20] proposed an approximation to the Choquet integral criteria aggregation that did not require ordering. By extending Marichal’s concept of entropy for fuzzy measures, Liu et al. [21] proposed a new method for determining fuzzy measures of the Choquet integral. Wen et al. [22] introduced Choquet integral-based linguistic operators under fuzzy heterogeneous environments for supplier selection in supply chain management. Some scholars also extended the Choquet integral to other fuzzy environments, such as in interval intuitionistic fuzzy information [23], the dual hesitant fuzzy environment [24], the interval-valued intuitionistic hesitant fuzzy environment [19] and the Pythagorean fuzzy environment [25]. Point operators are another aggregation tool to reduce the uncertainty of the aggregated arguments and thus obtain intensive information in the process of decision making. Since the point operator was proposed [26], it has been applied to many fields and has attracted increasing attention. Liu and Wang [27] proposed some point operators to translate IFS into another one. Xia and Xu [28] used the point operators to propose some operators for aggregating intuitionistic fuzzy values, and further extended those operators to intuitionistic multiplicative sets [29]. Peng [30,31], and Xing [32] also extended point operators to Pythagorean fuzzy sets, interval-valued Pythagorean fuzzy sets, and dual hesitant fuzzy sets, respectively.

However, the medical diagnosis problem in the real world is complex than many other applications. For instance: (1) We need to exactly express fuzzy information, and picture fuzzy numbers (PFNs) can depict doctors’ diagnoses for patients with respect to the symptoms; (2) We need to consider correlations among symptoms, and then the Choquet integral operator can be utilized to solve this problem; and (3) We need to reduce the uncertainty of doctor’s diagnosis data and get intensive information when diagnosing diseases. We can select point operators to achieve this function by adjusting the degree of doctor’s diagnosis data with some parameters. In order to solve above problems simultaneously, it is necessary to combine point operator with Choquet integral operator under picture fuzzy environment. Thus, the goal of this paper is to establish a new decision-making method that can not only control the certainty of doctor’s diagnosis data, but also deal with these situations where the diagnosis data are correlative. Then we apply new decision-making method to judge patient condition, and patients with different conditions are divided into different levels of hospitals instead of all patients rushing to large hospitals.

The rest of this paper is organized as follows. In the following section, we review some basic concepts related to PFS and the Choquet integral. In Section 3, we define some picture fuzzy point operators. In Section 4, by combining the point operators with Choquet integral operator, we propose the picture fuzzy point–Choquet averaging (PFPCA) operator, the picture fuzzy point–Choquet geometric (PFPCG) operator, the generalized picture fuzzy point–Choquet averaging (GPFPCA) operator and the generalized picture fuzzy point–Choquet geometric (GPFPCG) operator. Some prominent properties and special cases of these proposed operators are also studied. In Section 5, we introduce a novel method for solving MADM with picture fuzzy information based on the proposed operators. In Section 6, we provide an application example about assisting the hierarchical medical system to show the performance of new method.

## 2. Preliminaries

In the section, we briefly review some basic notions including PFS and the Choquet integral.

### 2.1. Picture Fuzzy Sets

**Definition** **1** [8]**.** *Let X be an ordinary fixed set; then a picture fuzzy set P defined on X is given by*

(1) $$P=\left\{\langle x,\mu_{p}\left(x\right),\eta_{p}\left(x\right),v_{p}\left(x\right)\rangle |x\in X\right\},$$

*where* $\mu_{p}\left(x\right)$ *is the positive degree of* x∈X*, and* $\eta_{p}\left(x\right)$ *and* $v_{p}\left(x\right)$ *are the neutral degree and negative degree, respectively, satisfying*

(2) $$\mu_{p}\left(x\right)+\eta_{p}\left(x\right)+v_{p}\left(x\right)\leq 1.$$

The uncertainty associated with PFS $\pi_{P}\left(x\right)=1-\mu_{p}\left(x\right)-\eta_{p}\left(x\right)-v_{p}\left(x\right)$ is also defined. In the case $\eta_{p}\left(x\right)=0,$ PFS is reduced to the IFS, and when both $\mu_{p}\left(x\right),v_{p}\left(x\right)=0$, PFS is reduced to the fuzzy set.

For simplicity, we use the pair (μ(x),η(x),v(x)) to denote a general PFN that can be denoted by p=(μ,η,v).

Given three PFNs $p=\left(\mu ,\eta ,v\right),p_{1}=\left(\mu_{1},\eta_{1},v_{1}\right),p_{1}=\left(\mu_{2},\eta_{2},v_{2}\right),$ Cuong [8] defined the operations of intersection, union, complement and inclusion for them, which can be described as below:

(3) $$p_{1}\cap p_{2}=\left(min\left(\mu_{1},\mu_{2}\right),max\left(\eta_{1},\eta_{2}\right),max\left(v_{1},v_{2}\right)\right),$$

(4) $$p_{1}\cup p_{2}=\left(max\left(\mu_{1},\mu_{2}\right),min\left(\eta_{1},\eta_{2}\right),min\left(v_{1},v_{2}\right)\right),$$

(5) $$p^{c}=\left(v,\eta ,\mu \right),$$

(6) $$p_{1}\subseteq p_{2},\;\mathrm{if}\;\mu_{1}\leq \mu_{2},\eta_{1}\leq \eta_{2}\;\mathrm{and}\;v_{1}\geq v_{2}.$$

Wei [9] further defines some operational laws for PFNs as shown below:

(7) $$p_{1}⊕p_{2}=\left(\left(\mu_{1}+\mu_{2}-\mu_{1}\mu_{2}\right),\eta_{1}\eta_{2},v_{1}v_{2}\right),$$

(8) $$p_{1}⊗p_{2}=\left(\mu_{1}\mu_{2},\eta_{1}+\eta_{2}-\eta_{1}\eta_{2},v_{1}+v_{2}-v_{1}v_{2}\right),$$

(9) $$\lambda p=\left(1-{\left(1-\mu \right)}^{\lambda },\eta^{\lambda },v^{\lambda }\right),$$

(10) $$p^{\lambda }=\left(\mu^{\lambda },1-{\left(1-\eta \right)}^{\lambda },1-{\left(1-v\right)}^{\lambda }\right).$$

**Definition** **2** [13]**.** *For two PFNs* $p_{1}=\left(\mu_{1},\eta_{1},v_{1}\right),p_{2}=\left(\mu_{2},\eta_{2},v_{2}\right),$ *their relations are defined as follows:*

(11) $$p_{1}\geq p_{2}\;\mathrm{iff}\;\forall x∊X,\mu_{1}\geq \mu_{2},v_{1}\leq v_{2},$$

(12) $$p_{1}=p_{2}\;\mathrm{iff}\;\forall x∊X,\mu_{1}=\mu_{2},v_{1}=v_{2}.$$

In order to rank the PFNs, Garg [13] gave the score function and accuracy function of PFNs.

**Definition** **3** [13]**.** *Suppose that* p=(μ,η,v) *is a PFN; then the score function of* p *is shown as follows:*

(13) $$S_{p}=\mu_{p}-v_{p}.$$

**Definition** **4** [13]**.** *Suppose that* p=(μ,η,v) *is a PFN; then the accuracy function of* p *is shown as follows:*

(14) $$H_{p}=\mu_{p}+\eta_{p}+v_{p}.$$

Based on the score and accuracy function of PFN, Garg further defines the following ranking rules to compare two PFNs.

**Definition** **5.** *For two PFNs:*

(15) $$\mathrm{if}\;S_{p_{1}}>S_{p_{2}},\;\mathrm{then}\;p_{1}>p_{2},$$

(16) $$\mathrm{if}\;S_{p_{1}}=S_{p_{2}},\;\mathrm{then}$$

$$\mathrm{if}\;H_{p_{1}}>H_{p_{2}},\;then\;p_{1}>p_{2},$$

$$\mathrm{if}\;H_{p_{1}}=H_{p_{2}},\;then\;p_{1}=p_{2}.$$

### 2.2. Choquet Integral Operator

The fuzzy measure can be used to define a weight on each combination of criteria in the Choquet integral model. In this subsection, we introduce the definitions of fuzzy measure and Choquet integral.

**Definition** **6** [33]**.** *A fuzzy measure on X is a set function* ρ:Γ(x)→[0,1], *with the following conditions:*

*(1)*  ρ(ϕ)=0,ρ(X)=1*(boundary conditions),*
*(2)*  A,B∈X*and*A⊆B*, then*ρ(A)≤ρ(B)*(monotonicity).*

However, we generally need to determine $2^{n}-2$ values for *n* criteria, which is quite complex, and thus it is not easy to give such fuzzy measure according to Definition 6. Therefore, the following σ-fuzzy measure ρ is further defined:

(17) ρ(A∪B) = ρ(A)+ρ(B)+σρ(A)ρ(B),

where A∪B=ϕ, and the parameter σ∈[−1,+∞) denotes the interaction between attributes. In Equation (17):

(1) If σ = 0, then σ-fuzzy measure ρ reduces to ρ(A∪B)=ρ(A)+ρ(B),A∪B=ϕ, which is defined as an additive measure.
  In this situation, if all the elements in X are independent, we get
  (18) $$\rho \left(A\right)=\sum_{x_{i}\in A}\rho \left(x_{i}\right).$$
(2) If all the elements in X are finite, then
  (19) $$\rho \left(A\right)=\rho \left(\overset{n}{\underset{i=1}{\cup }}x_{i}\right)=\{\begin{array}{cc} \frac{1}{\sigma }\left[\prod_{i=1}^{n}\left(1+\sigma \rho \left(x_{i}\right)\right)-1\right], & \sigma \neq 0\\ \sum_{x_{i}\in A}\rho \left(x_{i}\right), & \sigma =0 \end{array},$$
  where $x_{i}\cap x_{j}=\Phi$, for i,j=1,2⋯n, and i≠j.
(3) If ρ≻0, then σ-fuzzy measure ρ reduces to ρ(A∪B)≻ρ(A)+ρ(B), which is defined as a super-additive measure.
(4) If −1≤ρ≺0, then σ-fuzzy measure ρ reduces to ρ(A∪B)≺ρ(A)+ρ(B), which is defined as a sub-additive measure.

When using a fuzzy measure to model the importance of decision criteria set S, a well-known aggregation function is the Choquet integral [16].

**Definition** **7.** *Let* f *be a positive real-valued function on* X *and* ρ *be a fuzzy measure on* X *. The discrete Choquet integral of* f *with respect to* ρ *is defined as*

(20) $$\left(C\right)\int fd\rho =\sum_{i=1}^{n}\left[\rho \left(A_{\sigma \left(i\right)}\right)-\rho \left(A_{\sigma \left(i-1\right)}\right)\right]f_{\sigma \left(i\right)},$$

*where* σ(i) *denotes a permutation of* (1,2⋯n) *such that* $f_{\sigma \left(1\right)}\geq f_{\sigma \left(2\right)}\geq \cdots \geq f_{\sigma \left(n\right)},$ *and* $A_{\sigma \left(0\right)}=\phi ,$ $A_{\sigma \left(i\right)}=\{x_{\sigma \left(1\right)},\cdots x_{\sigma \left(i\right)}\}.$

## 3. Some Point Operations for Picture Fuzzy Numbers and Their Properties

Motivated by the idea of intuitionistic fuzzy point operators [28] and dual hesitant fuzzy point operators [32], we will define a series of picture fuzzy point operations to obtain more intensive information and further analyze some desirable properties of these operations, which are very useful in the remainder of this paper.

**Definition** **8.** *For a PFN*p=(μ,η,v)*, let*α,β,γ∈[0,1],*we define some PF point operators: PFN* ⟶ *PFN as follows:*

(21) $$D_{\alpha ,\beta }^{}\left(p\right)=\left\{\mu_{p}+\alpha \pi_{p},\eta_{p}+\beta \pi_{p},v_{p}+\left(1-\alpha -\beta \right)\pi_{p}\right\},$$

(22) $$F_{\alpha ,\beta ,\gamma }\left(p\right)=\left\{\mu_{p}+\alpha \pi_{p},\eta_{p}+\beta \pi_{p},v_{p}+\gamma \pi_{p}\right\},$$

*where* α+β+γ≤1

(23) $$G_{\alpha ,\beta ,\gamma }\left(p\right)=\left\{\alpha \mu_{p},\beta \eta_{p},\gamma v_{p}\right\}.$$

It is obvious that the above PF point operators transform a PFN into another one. From Equations (21) and (22), we know that $D_{\alpha }\left(p\right)$ assigns all the uncertainty into the other three parts of a PFS, while $F_{\alpha ,\beta ,\gamma }\left(p\right)$ only assigns part of the uncertainty. Meanwhile, we can get $\pi_{D_{\alpha }\left(p\right)}=1-\pi_{p}$, and $\pi_{F_{\alpha ,\beta ,\gamma }\left(p\right)}=\pi_{p}\left(1-\alpha -\beta -\gamma \right)$, which means that $F_{\alpha ,\beta ,\gamma }\left(p\right)$ and $D_{\alpha }\left(p\right)$ can reduce the uncertainty of PFS, and increase the positive degree, neutral degree, and positive degree. Similarly, From Equation (23), we know that $G_{\alpha ,\beta ,\gamma }\left(p\right)$ can reduce the positive degree, neutral degree, and positive degree, and $\pi_{G_{\alpha ,\beta ,\gamma }}=\left(1-\alpha \mu_{p}^{}-\beta \eta_{p}-\gamma v_{p}^{}\right)$, which means that $G_{\alpha ,\beta ,\gamma }\left(p\right)$ increases the uncertainty of PFS.

Then, we discuss some properties of the operator $F_{\alpha ,\beta ,\gamma }\left(p\right)$ in detail.

**Theorem** **1.** *Let* p=(μ,η,v) *be a PFN and taking* α,β,γ∈[0,1] *, then*

(24) $${\left(F_{\alpha ,\beta ,\gamma }\left(p^{c}\right)\right)}^{c}=F_{\gamma ,\beta ,\alpha }\left(p\right),$$

(25) $${\left(G_{\alpha ,\beta ,\gamma }\left(p^{c}\right)\right)}^{c}=G_{\gamma ,\beta ,\alpha }\left(p\right).$$

*If*

(26) $$\alpha =\frac{\mu_{p}}{\mu_{p}+\eta_{p}+v_{p}},\;\beta =\frac{\eta_{p}}{\mu_{p}+\eta_{p}+v_{p}}\;\mathrm{and}\;\gamma =\frac{v_{p}}{\mu_{p}+\eta_{p}+v_{p}}\;\mathrm{then}\;F_{\alpha ,\beta ,\gamma }\left(p\right)=\left(\alpha ,\beta ,\gamma \right).$$

**Proof.**  We prove the Equation (24) holds, and (25), (26) can be proved analogously.

(1) From $p^{c}=\left(v,\eta ,\mu \right)$, we get
  $${\left(F_{\alpha ,\beta ,\gamma }\left(p^{c}\right)\right)}^{c}={\left(v_{p}+\alpha \pi_{p},\eta_{p}+\beta \pi_{p},\mu_{p}+\gamma \pi_{p}\right)}^{c}=F_{\gamma ,\beta ,\alpha }\left(p\right).$$
(2) Then
  $$\begin{array}{ll} F_{\alpha ,\beta ,\gamma }\left(p\right) & =\left(\mu_{p}+\frac{\mu_{p}}{\mu_{p}+\eta_{p}+v_{p}}\pi_{p},\eta_{p}+\frac{\eta_{p}}{\mu_{p}+\eta_{p}+v_{p}}\pi_{p},v_{p}+\frac{v_{p}}{\mu_{p}+\eta_{p}+v_{p}}\pi_{p}\right)\\ & =\left(\frac{\mu_{p}}{\mu_{p}+\eta_{p}+v_{p}},\frac{\eta_{p}}{\mu_{p}+\eta_{p}+v_{p}},\frac{v_{p}}{\mu_{p}+\eta_{p}+v_{p}}\right)=\left(\alpha ,\beta ,\gamma \right) \end{array}.$$

Based on the operations of the PFNs, let $D_{\alpha }^{0}\left(p\right)=F_{\alpha ,\beta ,\gamma }^{0}\left(p\right)=G_{\alpha ,\beta ,\gamma }^{0}\left(p\right)=H_{\alpha ,\beta ,\gamma }^{0}\left(p\right)=p$; we then get the following Theorem 2. ☐

**Theorem** **2.** *Let* p=(μ,η,v) *be a PFN and taking* α,β,γ∈[0,1], *and* α+β+γ≠0, *then*

(27) $$D_{\alpha }^{n}\left(\gamma \right)=\left\{\mu_{p}+\alpha \pi_{p},\eta_{p}+\beta \pi_{p},v_{p}+\left(1-\alpha -\beta \right)\pi_{p}\right\},$$

(28) $$F_{\alpha ,\beta ,\gamma }^{n}\left(p\right)=\left(\mu_{p}+\alpha \pi_{p}\tau ,\eta_{p}+\beta \pi_{p}\tau ,v_{p}+\gamma \pi_{p}\tau \right),$$

*where* $\tau =\frac{1-{\left(1-\alpha -\beta -\gamma \right)}^{n}}{\alpha +\beta +\gamma }$ *,*

(29) $$G_{\alpha ,\beta ,\gamma }^{n}\left(p\right)=\left(\mu_{p}\alpha^{n},\eta_{p}\beta^{n},v_{p}\gamma^{n}\right).$$

The proof of this theorem is provided in Appendix A.

In the following, a numeric example is forwarded to illustrate Theorems 1 and 2.

**Example** **1.** *Let* p=(0.15, 0.35, 0.25) *be a PFN, then the point operators of p can be calculated according to Definition 8 (Suppose* α=0.4, β=0.3, γ=0.2 *). Firstly, we can obtain* $\pi_{p}=1-(0.15+0.35+0.25)=0.25$ *, and* $\tau =\frac{1-{\left(1-\alpha -\beta -\gamma \right)}^{n}}{\alpha +\beta +\gamma }=\frac{1-0.1^{n}}{0.9},$ *then we have*

(30) $$D_{\alpha ,\beta }^{}\left(p\right)=\left\{0.15+0.25\alpha ,0.35+0.25\beta ,0.25+0.25\left(1-\alpha -\beta \right)\right\}=\left(0.25,\;0.425,\;0.325\right),$$

(31) $$F_{\alpha ,\beta ,\gamma }\left(p\right)=\left(0.15+0.25\alpha ,0.35+0.25\beta ,0.25+0.25\gamma \right)=\left(0.25,\;0.425,\;0.3\right),$$

(32) $$G_{\alpha ,\beta ,\gamma }\left(p\right)=\left(0.15\alpha ,0.35\beta ,0.25\gamma \right)=\left(0.06,\;0.105,\;0.05\right).$$

*Similarly,*

(33) $$D_{\alpha }^{n}\left(\gamma \right)=\left(0.25,\;0.425,\;0.325\right),$$

(34) $$F_{\alpha ,\beta ,\gamma }^{n}\left(p\right)=\left(0.15+\frac{1-0.1^{n}}{9},0.35+\frac{0.75\times \left(1-0.1^{n}\right)}{9},0.25+\frac{5\times \left(1-0.1^{n}\right)}{9}\right),$$

(35) $$G_{\alpha ,\beta ,\gamma }^{n}\left(p\right)=\left\{0.15\times 0.4^{n},0.35\times 0.3^{n},0.25\times 0.2^{n}\right\}.$$

From Theorem 2, we can easily obtain the following properties.

**Theorem** **3.** *Let* p=(μ,η,v) *be a PFS, and n be a positive integer. Taking* α,β,γ∈[0,1], *then*

(36) $${\left(F_{\alpha ,\beta ,\gamma }^{n}\left(p^{c}\right)\right)}^{c}=F_{\gamma ,\beta ,\alpha }^{n}\left(p\right),$$

(37) $${\left(G_{\alpha ,\beta ,\gamma }^{n}\left(p^{c}\right)\right)}^{c}=G_{\gamma ,\beta ,\alpha }^{n}\left(p\right).$$

**Theorem** **4.** *Let* p=(μ,η,v) *be a PFS, and n be a positive integer. Taking* α,β,γ∈[0,1], *the relation* ≤ *is defined as* A≤B *if and only if* $\mu_{F_{\alpha ,\beta ,\gamma }^{n}\left(p\right)}\leq \mu_{F_{\alpha ,\beta ,\gamma }^{n-1}\left(p\right)},$ *and* $v_{F_{\alpha ,\beta ,\gamma }^{n}\left(p\right)}\leq v_{F_{\alpha ,\beta ,\gamma }^{n-1}\left(p\right)},$ *and then*

(38) $${\left(F_{\alpha ,\beta ,\gamma }^{n}\left(p^{c}\right)\right)}^{c}=F_{\gamma ,\beta ,\alpha }^{n}\left(p\right),$$

(39) $$\pi_{F_{\alpha ,\beta ,\gamma }^{n}}\leq \pi_{F_{\alpha ,\beta ,\gamma }^{n-1}}.$$

(40) $$\mathrm{If}\;\alpha =\frac{\mu_{p}}{\mu_{p}+\eta_{p}+v_{p}},\beta =\frac{\eta_{p}}{\mu_{p}+\eta_{p}+v_{p}},\gamma =\frac{v_{p}}{\mu_{p}+\eta_{p}+v_{p}},\;\mathrm{then}\;F_{\alpha ,\beta ,\gamma }^{n}\left(p\right)=F_{\alpha ,\beta ,\gamma }\left(\gamma \right).$$

**Definition** **9.** *Let* α,β,γ∈[0,1], *and α + β + γ ≤ 1. We define the following limit:*

(41) $$\underset{n\to \infty }{\lim}F_{\alpha ,\beta ,\gamma }^{n}\left(p\right)=\underset{n\to \infty }{\lim}\left\{\mu_{F_{\alpha ,\beta ,\gamma }^{n}\left(p\right)},\eta_{F_{\alpha ,\beta ,\gamma }^{n}\left(p\right)},v_{F_{\alpha ,\beta ,\gamma }^{n}\left(p\right)}\right\}.$$

**Theorem** **5.** *Let* α,β,γ∈[0,1], *and* α+β+γ≤1 *; then we have*

(42) $$\underset{n\to \infty }{\lim}F_{\alpha ,\beta ,\gamma }^{n}\left(p\right)==D_{\frac{\alpha }{\alpha +\beta +\gamma },\frac{\beta }{\alpha +\beta +\gamma }}^{n}\left(p\right).$$

**Proof of Theorem** **5.** According to Theorem 7, we get

$$\underset{n\to \infty }{\lim}\mu_{F_{\xi \zeta \left(\gamma \right)}^{n}}=\underset{n\to \infty }{\lim}\left(\mu_{p}+\alpha \pi_{p}\frac{1-{\left(1-\alpha -\beta -\gamma \right)}^{n}}{\alpha +\beta +\gamma }\right)=\mu_{p}+\frac{\alpha }{\alpha +\beta +\gamma }\pi_{p},$$

$$\underset{n\to \infty }{\lim}\eta_{F_{\xi \zeta \left(\gamma \right)}^{n}}=\underset{n\to \infty }{\lim}\left(\eta_{p}+\beta \pi_{p}\frac{1-{\left(1-\alpha -\beta -\gamma \right)}^{n}}{\alpha +\beta +\gamma }\right)=\eta_{p}+\frac{\beta }{\alpha +\beta +\gamma }\pi_{p},$$

$$\underset{n\to \infty }{\lim}v_{F_{\xi \zeta \left(\gamma \right)}^{n}}==v_{p}+\frac{\gamma }{\alpha +\beta +\gamma }\pi_{p}.$$

So we have

$$\underset{n\to \infty }{\lim}F_{\alpha ,\beta ,\gamma }^{n}\left(p\right)=\underset{n\to \infty }{\lim}\left\{\mu_{F_{\alpha ,\beta ,\gamma }^{n}\left(p\right)},\eta_{F_{\alpha ,\beta ,\gamma }^{n}\left(p\right)},v_{F_{\alpha ,\beta ,\gamma }^{n}\left(p\right)}\right\}\;=\left\{\mu_{p}+\frac{\alpha }{\alpha +\beta +\gamma }\pi_{p},\eta_{p}+\frac{\beta }{\alpha +\beta +\gamma }\pi_{p},v_{p}+\frac{\gamma }{\alpha +\beta +\gamma }\pi_{p}\right\}=D_{\frac{\alpha }{\alpha +\beta +\gamma },\frac{\beta }{\alpha +\beta +\gamma }}^{n}\left(p\right)$$.

 ☐

## 4. Picture Fuzzy Point–Choquet Integral Aggregation Operators and Their Properties

In order to get more intensive information from PFS and efficiently deal with correlations among arguments at the same time, we combine picture fuzzy point operators with the Choquet integral operator to propose some new class of aggregation operators for aggregating picture fuzzy information in this section. Some desirable properties of proposed aggregation operators are also discussed in detail.

### 4.1. Picture Fuzzy Point–Choquet Averaging Operator

**Definition** **10.** *Let* Ω *be the set of all PFNs, and* $p_{i}=\left(\mu_{i},\eta_{i},v_{i}\right)\left(i=1,2,\ldots ,m\right)$ *be a collection of PFNs, taking* $\alpha_{i},\beta_{i},\gamma_{i}\in \left[0,1\right].$ *Then we define the series of PFPCA operators):* $\Omega^{m}\to \Omega$ *, if*

(43) $$F\left(C_{1}\right)\int pd\rho =PFPCAD_{\alpha ,\beta }^{n}\left(p_{1},p_{2}\cdots p_{n}\right)=\sum_{i=1}^{m}\left(\rho \left(A_{\sigma \left(i\right)}\right)-\rho \left(A_{\sigma \left(i-1\right)}\right)\right)D_{\alpha_{\sigma \left(i\right),}\beta_{\sigma \left(i\right)}}^{n}\left(p_{\sigma \left(i\right)}\right),$$

(44) $$F\left(C_{2}\right)\int pd\rho =PFPCAF_{\alpha_{,}\beta ,\gamma }^{n}\left(p_{1},p_{2}\cdots p_{n}\right)=\sum_{i=1}^{m}\left(\rho \left(A_{\sigma \left(i\right)}\right)-\rho \left(A_{\sigma \left(i-1\right)}\right)\right)F_{\alpha_{\sigma \left(i\right),}\beta_{\sigma \left(i\right)},\gamma_{\sigma \left(i\right)}}^{n}\left(p_{\sigma \left(i\right)}\right),$$

(45) $$F\left(C_{3}\right)\int pd\rho =PFPCAG_{\alpha_{,}\beta ,\gamma }^{n}\left(p_{1},p_{2}\cdots p_{n}\right)=\sum_{i=1}^{m}\left(\rho \left(A_{\sigma \left(i\right)}\right)-\rho \left(A_{\sigma \left(i-1\right)}\right)\right)G_{\alpha_{\sigma \left(i\right),}\beta_{\sigma \left(i\right)},\gamma_{\sigma \left(i\right)}}^{n}\left(p_{\sigma \left(i\right)}\right),$$

*where* σ(i) *denotes a permutation of* (1,2⋯m) *such that* $p_{\sigma \left(1\right)}\geq p_{\sigma \left(2\right)}\geq \cdots \geq p_{\sigma \left(m\right)},$ *and* $G_{\sigma \left(i\right)}$ *is the attribute corresponding to* $p_{\sigma \left(i\right)}.$

By operational laws defined in Section 2.1, we can obtain the following theorem.

**Theorem** **6.** *Let* $p_{i}=\left(\mu_{i},\eta_{i},v_{i}\right)\left(i=1,2,\ldots ,m\right)$ *be a collection of PFNs, and* σ(i) *be a permutation of* (1,2⋯m) *such that* $p_{\sigma \left(1\right)}\geq p_{\sigma \left(2\right)}\geq \cdots \geq p_{\sigma \left(m\right)},$ $G_{\sigma \left(i\right)}$ *is the attribute corresponding to* $p_{\sigma \left(i\right)},$ *and* $A_{\sigma \left(0\right)}=\phi$ $A_{\sigma \left(i\right)}=\{G_{\sigma \left(1\right)},\cdots G_{\sigma \left(i\right)}\},$ *taking* ${\tilde{\omega }}_{i}=\rho \left(A_{\sigma \left(i\right)}\right)-\rho \left(A_{\sigma \left(i-1\right)}\right),$ $\alpha_{i},\beta_{i},\gamma_{i}\in \left[0,1\right],$ $\alpha_{i}+\beta_{i}+\gamma_{i}\leq 1$ *. Then, the aggregated values by the series of PFPCA operators are also PFNs:*

(46) $$\begin{array}{ll} PFPCAD_{\alpha ,\beta }^{n}\left(p_{1},p_{2}\cdots p_{n}\right) & =\{1-\prod_{i=1}^{m}{\left(1-\left(\mu_{p_{\sigma \left(i\right)}}+\alpha_{i}\pi_{p_{\sigma \left(i\right)}}\right)\right)}^{{\tilde{\omega }}_{i}},1-\prod_{i=1}^{m}{\left(1-\left(\eta_{p_{\sigma \left(i\right)}}+\beta_{i}\pi_{p_{\sigma \left(i\right)}}\right)\right)}^{{\tilde{\omega }}_{i}}\\ & \prod_{i=1}^{m}{\left(v_{p_{\sigma \left(i\right)}}+\left(1-\alpha_{i}-\beta_{i}\right)\pi_{p_{\sigma \left(i\right)}}\right)}^{{\tilde{\omega }}_{i}}\} \end{array},$$

(47) $$PFPCAF_{\alpha ,\beta ,\gamma }^{n}\left(p_{1},p_{2},\ldots ,p_{m}\right)=\left(\left(1-\prod_{i=1}^{m}{\left(1-\mu_{F_{\alpha_{i},\beta_{i},\gamma_{i}}^{n}\left(p_{\sigma \left(i\right)}\right)}\right)}^{{\tilde{\omega }}_{i}}\right),\prod_{i=1}^{m}\eta_{F_{\alpha_{i},\beta_{i},\gamma_{i}}^{n}\left(p_{\sigma \left(i\right)}\right)}^{{\tilde{\omega }}_{i}},\prod_{i=1}^{m}v_{F_{\alpha_{i},\beta_{i},\gamma_{i}}^{n}\left(p_{\sigma \left(i\right)}\right)}^{{\tilde{\omega }}_{i}}\right)$$

*where*

$$\mu_{F_{\alpha_{i},\beta_{i},\gamma_{i}}^{n}\left(p_{i}\right)}=\mu_{p_{\sigma \left(i\right)}}+\alpha_{i}\pi_{p_{\sigma \left(i\right)}}\frac{1-{\left(1-\alpha_{i}-\beta_{i}-\gamma_{i}\right)}^{n}}{\alpha_{i}+\beta_{i}+\gamma_{i}},$$

$$\eta_{F_{\alpha_{i},\beta_{i},\gamma_{i}}^{n}\left(p_{i}\right)}=\eta_{p_{\sigma \left(i\right)}}+\beta_{i}\pi_{p_{\sigma \left(i\right)}}\frac{1-{\left(1-\alpha_{i}-\beta_{i}-\gamma_{i}\right)}^{n}}{\alpha_{i}+\beta_{i}+\gamma_{i}},$$

$$v_{F_{\alpha_{i},\beta_{i},\gamma_{i}}^{n}\left(p_{\sigma \left(i\right)}\right)}=v_{p_{\sigma \left(i\right)}}+\gamma_{i}\pi_{p_{\sigma \left(i\right)}}\frac{1-{\left(1-\alpha_{i}-\beta_{i}-\gamma_{i}\right)}^{n}}{\alpha_{i}+\beta_{i}+\gamma_{i}},$$

(48) $$PFPCAG_{\alpha ,\beta ,\gamma }^{n}\left(p_{1},p_{2},\ldots ,p_{m}\right)=\left(\left(1-\prod_{i=1}^{m}{\left(1-\mu_{p_{\sigma \left(i\right)}}\alpha_{i}^{n}\right)}^{{\tilde{\omega }}_{i}}\right),\prod_{i=1}^{m}{\left(\eta_{p_{\sigma \left(i\right)}}\beta_{i}^{n}\right)}^{{\tilde{\omega }}_{i}},\prod_{i=1}^{m}{\left(v_{p_{\sigma \left(i\right)}}\gamma_{i}^{n}\right)}^{{\tilde{\omega }}_{i}}\right).$$

The proof of this theorem is provided in Appendix B.

In the following, a numeric example is forwarded to illustrate Theorem 6.

**Example** **2.** *Let* $p_{1}=\left(0.25,\;0.35,\;0.15\right)$ *,* $p_{2}=\left(0.42,\;0.18,\;0.37\right),$ $p_{3}=\left(0.34,\;0.27,\;0.16\right)$ *be PFN. Then we aggregate the three PFNs by the following steps:*

**Step 1.** Identify the fuzzy measure of the n attributes of G according to Equations (17) and (19). Suppose that the fuzzy measures of attributes of G are given as follows:

$$\rho \left(G_{1}\right)=0.38,\rho \left(G_{2}\right)=0.27,\rho \left(G_{3}\right)=0.36.$$

Firstly, according to Equation (19), the value of σ is obtained: σ=−0.029, and then the fuzzy measures of attribute sets of $G=\{G_{1},G_{2},G_{3},G_{4}\}$ can be calculated by Equation (13), shown as follows:

$$\rho \left(G_{1},G_{2}\right)=0.65,\rho \left(G_{1},G_{3}\right)=0.74,\;\rho \left(G_{2},G_{3}\right)=0.63,\;\rho \left(G_{1},G_{2},G_{3}\right)=1.$$

**Step 2.** By score functions, we rearrange the three PFNs in descending order, shown as follows:

$$s\left(p_{1}\right)=0.1,s\left(p_{2}\right)=0.05,s\left(p_{3}\right)=0.18,$$

$$p_{\sigma \left(1\right)}=\left(0.34,\;0.27,\;0.16\right),p_{\sigma \left(2\right)}=\left(0.25,\;0.35,\;0.15\right),p_{\sigma \left(3\right)}=\left(0.42,\;0.18,\;0.37\right).$$

Then we can get

$$A_{\sigma \left(1\right)}=\{G_{3}\},A_{1\sigma \left(2\right)}=\{G_{1},G_{3}\},A_{1\sigma \left(4\right)}=\{G_{1},G_{2},G_{3}\},$$

$$\rho_{A_{\sigma \left(1\right)}}=\rho_{G_{3}}=0.36,\rho_{A_{\sigma \left(2\right)}}-\rho_{A_{\sigma \left(1\right)}}=\rho_{G_{1}G_{3}}-\rho_{G_{3}}=0.38,\rho_{A_{\sigma \left(3\right)}}-\rho_{A_{\sigma \left(2\right)}}=\rho_{G_{2}G_{3}G_{4}}-\rho_{G_{1}G_{3}}=0.26.$$.

**Step 3.** Calculate the point operators of *p_i_* according to Definition 8 (Suppose α=0.3,β=0.4,γ=0.1,n=3). Firstly, we can obtain $\pi_{p_{\sigma \left(1\right)}}=1-(0.25+0.35+0.15)=0.25$, $\pi_{p_{\sigma \left(2\right)}}=1-(0.42+0.18+0.37)=0.03,$ $\pi_{p_{\sigma \left(3\right)}}=1-(0.34+0.27+0.16)=0.23$. Then we have

$$F_{\alpha ,\beta ,\gamma }^{3}\left(p_{\sigma \left(1\right)}\right)=\left(0.25+0.25\times \frac{1-0.2^{3}}{0.8},0.35+0.25\times \frac{1-0.2^{3}}{0.8},0.15+0.25\times \frac{1-0.2^{3}}{0.8}\right)=\left(0.56,0.66,0.46\right),$$

$$F_{\alpha ,\beta ,\gamma }^{3}\left(p_{\sigma \left(2\right)}\right)=\left(0.42+0.03\times \frac{1-0.2^{3}}{0.8},0.18+0.03\times \frac{1-0.2^{3}}{0.8},0.37+0.03\times \frac{1-0.2^{3}}{0.8}\right)=\left(0.46,0.22,0.41\right),\;F_{\alpha ,\beta ,\gamma }^{3}\left(p_{\sigma \left(3\right)}\right)=\left(0.34+0.23\times \frac{1-0.2^{3}}{0.8},0.27+0.23\times \frac{1-0.2^{3}}{0.8},0.16+0.23\times \frac{1-0.2^{3}}{0.8}\right)=\left(0.63,0.56,0.45\right)$$

**Step 4.** Utilize the $PFPCAF_{\alpha ,\beta ,\gamma }^{n}\left(p_{1},p_{2},p_{3}\right)$ operator to aggregate the three PFNs and get the aggregated *p* as follows:

$$p=\sum_{i=1}^{m}\left(\rho \left(A_{\sigma \left(i\right)}\right)-\rho \left(A_{\sigma \left(i-1\right)}\right)\right)F_{\alpha_{i,}\beta_{i},\gamma_{i}}^{n}\left(p_{\sigma \left(i\right)}\right)\;=\left(\left(1-\prod_{i=1}^{m}{\left(1-\mu_{F_{\alpha_{i},\beta_{i},\gamma_{i}}^{n}\left(p_{\sigma \left(i\right)}\right)}\right)}^{\rho \left(A_{\sigma \left(i\right)}\right)-\rho \left(A_{\sigma \left(i-1\right)}\right)}\right),\prod_{i=1}^{m}{\left(\eta_{F_{\alpha_{i},\beta_{i},\gamma_{i}}^{n}\left(p_{\sigma \left(i\right)}\right)}^{}\right)}^{\rho \left(A_{\sigma \left(i\right)}\right)-\rho \left(A_{\sigma \left(i-1\right)}\right)},\prod_{i=1}^{m}{\left(v_{F_{\alpha_{i},\beta_{i},\gamma_{i}}^{n}\left(p_{\sigma \left(i\right)}\right)}^{}\right)}^{\rho \left(A_{\sigma \left(i\right)}\right)-\rho \left(A_{\sigma \left(i-1\right)}\right)}\right)\;=\left(\left(1-{\left(1-0.56\right)}^{0.36}\times {\left(1-0.46\right)}^{0.38}\times {\left(1-0.63\right)}^{0.26}\right),0.66^{0.36}\times 0.22^{0.38}\times 0.56^{0.26},0.46^{0.36}\times 0.41^{0.38}\times 0.45^{0.26}\right).$$

Example 2 gives a detailed portrait of the $PFPCAF_{\alpha ,\beta ,\gamma }^{n}\left(p_{1},p_{2},p_{3}\right)$ operator. It should be pointed out that the $PFPCAF_{\alpha ,\beta ,\gamma }^{n}\left(p_{1},p_{2},p_{3}\right)$ operator includes a reorder step and it is similar to the famous ordered weighted averaging (OWA) operator. In the following, we discuss some properties of the above PFPCA operators.

**Theorem** **7.** *Let* $p_{i}=\left(\mu_{i},\eta_{i},v_{i}\right)\left(i=1,2,\ldots ,m\right)$ *be a collection of PFNs. Taking* k≻0, *then*

(49) $$PFPCAD_{\alpha ,\beta }^{n}\left(kp_{1},kp_{2},\ldots ,kp_{m}\right)=kPFPCAD_{\alpha ,\beta }^{n}\left(p_{1},p_{2},\ldots ,p_{m}\right),$$

(50) $$PFPCAF_{\alpha ,\beta ,\gamma }^{n}\left(kp_{1},kp_{2},\ldots ,kp_{m}\right)=kPFPCAF_{\alpha ,\beta ,\gamma }^{n}\left(p_{1},p_{2},\ldots ,p_{m}\right),$$

(51) $$PFPCAG_{\alpha ,\beta ,\gamma }^{n}\left(kp_{1},kp_{2},\ldots ,kp_{m}\right)=kPFPCAG_{\alpha ,\beta ,\gamma }^{n}\left(p_{1},p_{2},\ldots ,p_{m}\right).$$

**Proof.**  We prove the Equation (50) holds for all *m*, and the others can be proved analogously.

By the operational law in Section 2.2, we have

$$kp_{i}=\left(1-{\left(1-\mu_{i}\right)}^{k},\eta_{i}{}^{k},v_{i}{}^{k}\right)\;$$

and

$$PFPCAF_{\alpha ,\beta ,\gamma }^{n}\left(kp_{1},kp_{2},\ldots ,kp_{m}\right)=\left(1-\prod_{i=1}^{m}{\left(1-\mu_{F_{\alpha_{i},\beta_{i},\gamma_{i}}^{n}\left(p_{\sigma \left(i\right)}\right)}\right)}^{k{\tilde{\omega }}_{i}},\prod_{i=1}^{m}\eta_{F_{\alpha_{i},\beta_{i},\gamma_{i}}^{n}\left(p_{i}\right)}^{k{\tilde{\omega }}_{i}},\prod_{i=1}^{m}v_{F_{\alpha_{i},\beta_{i},\gamma_{i}}^{n}\left(p_{\sigma \left(i\right)}\right)}^{k{\tilde{\omega }}_{i}}\right),$$

and hence

$$\begin{array}{ll} kPFPCAF_{\alpha ,\beta ,\gamma }^{n}\left(p_{1},p_{2},\ldots ,p_{m}\right)= & k\left(1-\prod_{i=1}^{m}{\left(1-\mu_{F_{\alpha_{i},\beta_{i},\gamma_{i}}^{n}\left(p_{\sigma \left(i\right)}\right)}\right)}^{{\tilde{\omega }}_{i}},\prod_{i=1}^{m}\eta_{F_{\alpha_{i},\beta_{i},\gamma_{i}}^{n}\left(p_{\sigma \left(i\right)}\right)}^{k{\tilde{\omega }}_{i}},\prod_{i=1}^{m}v_{F_{\alpha_{i},\beta_{i},\gamma_{i}}^{n}\left(p_{\sigma \left(i\right)}\right)}^{{\tilde{\omega }}_{i}}\right)\\ & \left(1-\prod_{i=1}^{m}{\left(1-\mu_{F_{\alpha_{i},\beta_{i},\gamma_{i}}^{n}\left(p_{\sigma \left(i\right)}\right)}\right)}^{k{\tilde{\omega }}_{i}},\prod_{i=1}^{m}\eta_{F_{\alpha_{i},\beta_{i},\gamma_{i}}^{n}\left(p_{\sigma \left(i\right)}\right)}^{k{\tilde{\omega }}_{i}},\prod_{i=1}^{m}v_{F_{\alpha_{i},\beta_{i},\gamma_{i}}^{n}\left(p_{\sigma \left(i\right)}\right)}^{k{\tilde{\omega }}_{i}}\right)\\ & =PFPCAF_{\alpha ,\beta ,\gamma }^{n}\left(kp_{1},kp_{2},\ldots ,kp_{m}\right) \end{array}.$$

Therefore, Equation (50) holds, which completes the proof. ☐

**Theorem** **8.** *Let p_i_ and q_i_ be two collections of PFNs, then*

(52) $$PFPCAD_{\alpha ,\beta }^{n}\left(p_{1}⊕q_{1},p_{2}⊕q_{2},\ldots ,p_{n}⊕q_{m}\right)\;=PFPCAD_{\alpha ,\beta }^{n}\left(p_{1},p_{2},\ldots ,p_{m}\right)⊕\cdots ⊕PFPCAD_{\alpha ,\beta }^{n}\left(q_{1},q_{2},\ldots ,q_{m}\right)$$

(53) $$PFPCAF_{\alpha ,\beta ,\gamma }^{n}\left(p_{1}⊕q_{1},p_{2}⊕q_{2},\ldots ,p_{n}⊕q_{m}\right)\;=PFPCAF_{\alpha ,\beta ,\gamma }^{n}\left(p_{1},p_{2},\ldots ,p_{m}\right)⊕\cdots ⊕PFPCAF_{\alpha ,\beta ,\gamma }^{n}\left(q_{1},q_{2},\ldots ,q_{m}\right)$$

(54) $$PFPCAG_{\alpha ,\beta ,\gamma }^{n}\left(p_{1}⊕q_{1},p_{2}⊕q_{2},\ldots ,p_{n}⊕q_{m}\right)\;=PFPCAG_{\alpha ,\beta ,\gamma }^{n}\left(p_{1},p_{2},\ldots ,p_{m}\right)⊕\cdots ⊕PFPCAG_{\alpha ,\beta ,\gamma }^{n}\left(q_{1},q_{2},\ldots ,q_{m}\right)$$

**Proof.**  We prove the Equation (53) holds for all *m*, and the others can be proved analogously.

By the operational law in Section 2.2, we have

$$p_{i}⊕q_{i}=\left(\mu_{p_{i}}+\mu_{q_{i}}-\mu_{p_{i}}\mu_{q_{i}},\eta_{p_{i}}\eta_{q_{i}},v_{p_{i}}v_{q_{i}}\right),$$

$$\begin{array}{l} PFPCAF_{\alpha ,\beta ,\gamma }^{n}\left(p_{1}⊕q_{1},p_{2}⊕q_{2},\ldots ,p_{n}⊕q_{m}\right)\\ =(\left(1-\prod_{i=1}^{m}{\left(1-\mu_{F_{\alpha_{i},\beta_{i},\gamma_{i}}^{n}\left(p_{{}_{\sigma \left(i\right)}}\right)}\right)}^{{\tilde{\omega }}_{i}}{\left(1-\mu_{F_{\alpha_{i},\beta_{i},\gamma_{i}}^{n}\left(q_{{}_{\sigma \left(i\right)}}\right)}\right)}^{{\tilde{\omega }}_{i}}\right),\prod_{i=1}^{m}\eta_{F_{\alpha_{i},\beta_{i},\gamma_{i}}^{n}\left(p_{{}_{\sigma \left(i\right)}}\right)}^{{\tilde{\omega }}_{i}}\eta_{F_{\alpha_{i},\beta_{i},\gamma_{i}}^{n}\left(q_{{}_{\sigma \left(i\right)}}\right)}^{{\tilde{\omega }}_{i}},\prod_{i=1}^{m}v_{F_{\alpha_{i},\beta_{i},\gamma_{i}}^{n}\left(p_{{}_{\sigma \left(i\right)}}\right)}^{{\tilde{\omega }}_{i}}v_{F_{\alpha_{i},\beta_{i},\gamma_{i}}^{n}\left(q_{{}_{\sigma \left(i\right)}}\right)}^{{\tilde{\omega }}_{i}}) \end{array},$$

$$\begin{array}{l} PFPCAF_{\alpha ,\beta ,\gamma }^{n}\left(p_{1},p_{2},\ldots ,p_{m}\right)⊕PFPCAF_{\alpha ,\beta ,\gamma }^{n}\left(q_{1},q_{2},\ldots ,q_{m}\right)\\ =(1-\prod_{i=1}^{m}{\left(1-\mu_{F_{\alpha_{i},\beta_{i},\gamma_{i}}^{n}\left(p_{{}_{\sigma \left(i\right)}}\right)}\right)}^{{\tilde{\omega }}_{i}}{\left(1-\mu_{F_{\alpha_{i},\beta_{i},\gamma_{i}}^{n}\left(q_{{}_{\sigma \left(i\right)}}\right)}\right)}^{{\tilde{\omega }}_{i}},\prod_{i=1}^{m}\eta_{F_{\alpha_{i},\beta_{i},\gamma_{i}}^{n}\left(p_{{}_{\sigma \left(i\right)}}\right)}^{{\tilde{\omega }}_{i}}\eta_{F_{\alpha_{i},\beta_{i},\gamma_{i}}^{n}\left(q_{{}_{\sigma \left(i\right)}}\right)}^{{\tilde{\omega }}_{i}},\prod_{i=1}^{m}\nu_{F_{\alpha_{i},\beta_{i},\gamma_{i}}^{n}\left(p_{{}_{\sigma \left(i\right)}}\right)}^{{\tilde{\omega }}_{i}}\nu_{F_{\alpha_{i},\beta_{i},\gamma_{i}}^{n}\left(q_{{}_{\sigma \left(i\right)}}\right)}^{{\tilde{\omega }}_{i}})\\ =PFPCAF_{\alpha ,\beta ,\gamma }^{n}\left(p_{1},p_{2},\ldots ,p_{m}\right)⊕PFPCAF_{\alpha ,\beta ,\gamma }^{n}\left(q_{1},q_{2},\ldots ,q_{m}\right) \end{array}.$$

Therefore, Equation (53) holds, which completes the proof. ☐

**Theorem** **9.** *(Idempotency). If* $p_{i}=\left(\mu_{i},\eta_{i},v_{i}\right)$ *are equal, i.e.,* $p_{i}=p=\left(\mu ,\eta ,v\right)$ *for all i, then*

(55) $$PFPCAD_{\alpha ,\beta }^{n}\left(p_{1},p_{2},\ldots ,p_{m}\right)=D_{\alpha ,\beta }^{n},$$

(56) $$PFPCAF_{\alpha ,\beta ,\gamma }^{n}\left(p_{1},p_{2},\ldots ,p_{m}\right)=F_{\alpha ,\beta ,\gamma }^{n},$$

(57) $$PFPCAG_{\alpha ,\beta ,\gamma }^{n}\left(p_{1},p_{2},\ldots ,p_{m}\right)=G_{\alpha ,\beta ,\gamma }^{n}.$$

**Proof.**  We prove the Equation (56) holds for all *m*, and the others can be proved analogously.

Since $p_{i}=p=\left(\mu ,\eta ,v\right)$ for all *i*, then

$$PFPCAF_{\alpha ,\beta ,\gamma }^{n}\left(p_{1},p_{2},\ldots ,p_{m}\right)=\left(\left(1-\prod_{i=1}^{m}{\left(1-\mu_{F_{\alpha_{i},\beta_{i},\gamma_{i}}^{n}\left(p_{\sigma \left(i\right)}\right)}\right)}^{{\tilde{\omega }}_{i}}\right),\prod_{i=1}^{m}\eta_{F_{\alpha_{i},\beta_{i},\gamma_{i}}^{n}\left(p_{\sigma \left(i\right)}\right)}^{{\tilde{\omega }}_{i}},\prod_{i=1}^{m}v_{F_{\alpha_{i},\beta_{i},\gamma_{i}}^{n}\left(p_{\sigma \left(i\right)}\right)}^{{\tilde{\omega }}_{i}}\right)\;=\left(1-{\left(1-\mu_{F_{\alpha_{i},\beta_{i},\gamma_{i}}^{n}\left(p\right)}\right)}^{\sum_{i=1}^{m}\omega_{i}},{\left(\eta_{F_{\alpha_{i},\beta_{i},\gamma_{i}}^{n}\left(p\right)}\right)}^{\sum_{i=1}^{m}\omega_{i}},{\left(v_{F_{\alpha_{i},\beta_{i},\gamma_{i}}^{n}\left(p\right)}\right)}^{\sum_{i=1}^{m}\omega_{i}}\right)=\left(1-\left(1-\mu_{F_{\alpha_{i},\beta_{i},\gamma_{i}}^{n}\left(p\right)}\right),\eta_{F_{\alpha_{i},\beta_{i},\gamma_{i}}^{n}\left(p\right)},v_{F_{\alpha_{i},\beta_{i},\gamma_{i}}^{n}\left(p\right)}\right)\;=\left(\mu_{F_{\alpha_{i},\beta_{i},\gamma_{i}}^{n}\left(p\right)},\eta_{F_{\alpha_{i},\beta_{i},\gamma_{i}}^{n}\left(p\right)},v_{F_{\alpha_{i},\beta_{i},\gamma_{i}}^{n}\left(p\right)}\right)=F_{\alpha ,\beta ,\gamma }^{n}.$$

 ☐

**Theorem** **10.** *(Monotonicity) Let* $p_{i}=\left(\mu_{p_{i}},\eta_{p_{i}},v_{p_{i}}\right)$ *and* $q_{i}=\left(\mu_{q_{i}},\eta_{q_{i}},v_{q_{i}}\right)\left(i=1,2,\ldots ,m\right)$ *be two collections of PFN. If* $\mu_{p_{i}}\leq \mu_{q_{i}},\eta_{p_{i}}\geq \eta_{q_{i}}$ *and* $v_{p_{i}}\geq v_{q_{i}}$ *holds for all i* (i=1,2,…,m) *, then*

(58) $$PFPCAD_{\alpha ,\beta }^{n}\left(p_{1},p_{2},\ldots ,p_{m}\right)\leq PFPCAD_{\alpha ,\beta }^{n}\left(q_{1},q_{2},\ldots ,q_{m}\right),$$

(59) $$PFPCAF_{\alpha ,\beta ,\gamma }^{n}\left(p_{1},p_{2},\ldots ,p_{m}\right)\leq PFPCAF_{\alpha ,\beta ,\gamma }^{n}\left(q_{1},q_{2},\ldots ,q_{m}\right),$$

(60) $$PFPCAG_{\alpha ,\beta ,\gamma }^{n}\left(p_{1},p_{2},\ldots ,p_{m}\right)\leq PFPCAG_{\alpha ,\beta ,\gamma }^{n}\left(q_{1},q_{2},\ldots ,q_{m}\right).$$

**Proof.**  We prove the Equation (59) holds for all *m*, and the others can be proved analogously.

By Theorem 6, we get

$$PFPCAF_{\alpha ,\beta ,\gamma }^{n}\left(p_{1},p_{2},\ldots ,p_{m}\right)=\left(\left(1-\prod_{i=1}^{m}{\left(1-\mu_{F_{\alpha_{i},\beta_{i},\gamma_{i}}^{n}\left(p_{\sigma \left(i\right)}\right)}\right)}^{{\tilde{\omega }}_{i}}\right),\prod_{i=1}^{m}\eta_{F_{\alpha_{i},\beta_{i},\gamma_{i}}^{n}\left(p_{\sigma \left(i\right)}\right)}^{{\tilde{\omega }}_{i}},\prod_{i=1}^{m}v_{F_{\alpha_{i},\beta_{i},\gamma_{i}}^{n}\left(p_{\sigma \left(i\right)}\right)}^{{\tilde{\omega }}_{i}}\right),\;PFPCAF_{\alpha ,\beta ,\gamma }^{n}\left(q_{1},q_{2},\ldots ,q_{m}\right)=\left(\left(1-\prod_{i=1}^{m}{\left(1-\mu_{F_{\alpha_{i},\beta_{i},\gamma_{i}}^{n}\left(q_{\sigma \left(i\right)}\right)}\right)}^{{\tilde{\omega }}_{i}}\right),\prod_{i=1}^{m}\eta_{F_{\alpha_{i},\beta_{i},\gamma_{i}}^{n}\left(q_{\sigma \left(i\right)}\right)}^{{\tilde{\omega }}_{i}},\prod_{i=1}^{m}v_{F_{\alpha_{i},\beta_{i},\gamma_{i}}^{n}\left(q_{\sigma \left(i\right)}\right)}^{{\tilde{\omega }}_{i}}\right).$$

Since $\mu_{p_{i}}\leq \mu_{q_{i}}$ and $v_{p_{i}}\geq v_{q_{i}}$, we can get

$$\left(1-\prod_{i=1}^{m}{\left(1-\mu_{F_{\alpha_{i},\beta_{i},\gamma_{i}}^{n}\left(p_{\sigma \left(i\right)}\right)}\right)}^{{\tilde{\omega }}_{i}}\right)\leq \left(1-\prod_{i=1}^{m}{\left(1-\mu_{F_{\alpha_{i},\beta_{i},\gamma_{i}}^{n}\left(q_{\sigma \left(i\right)}\right)}\right)}^{{\tilde{\omega }}_{i}}\right)\;$$

and

$$\prod_{i=1}^{m}\eta_{F_{\alpha_{i},\beta_{i},\gamma_{i}}^{n}\left(p_{\sigma \left(i\right)}\right)}^{{\tilde{\omega }}_{i}}\geq \prod_{i=1}^{m}\eta_{F_{\alpha_{i},\beta_{i},\gamma_{i}}^{n}\left(q_{\sigma \left(i\right)}\right)}^{{\tilde{\omega }}_{i}},\prod_{i=1}^{m}v_{F_{\alpha_{i},\beta_{i},\gamma_{i}}^{n}\left(p_{\sigma \left(i\right)}\right)}^{{\tilde{\omega }}_{i}}\geq \prod_{i=1}^{m}v_{F_{\alpha_{i},\beta_{i},\gamma_{i}}^{n}\left(q_{\sigma \left(i\right)}\right)}^{{\tilde{\omega }}_{i}}.$$

By Definition 6, we get $PFPCAF_{\alpha ,\beta ,\gamma }^{n}\left(p_{1},p_{2},\ldots ,p_{m}\right)\leq PFPCAF_{\alpha ,\beta ,\gamma }^{n}\left(q_{1},q_{2},\ldots ,q_{m}\right)$. ☐

**Theorem** **11.** *(Boundedness) Let* $p_{i}=\left(\mu_{i},\eta_{i},v_{i}\right)\left(i=1,2,\ldots ,m\right)$ *be a collection of PFNs, then*

(61) $$d_{D_{\alpha ,\beta }^{n}}^{-}\leq PFPCAD_{\alpha ,\beta }^{n}\leq d_{D_{\alpha ,\beta }^{n}}^{+},$$

(62) $$d_{F_{\alpha ,\beta ,\gamma }^{n}}^{-}\leq PFPCAF_{\alpha ,\beta ,\gamma }^{n}\left(p_{1},p_{2},\ldots ,p_{m}\right)\leq d_{F_{\alpha ,\beta ,\gamma }^{n}}^{+},$$

(63) $$d_{G_{\alpha ,\beta ,\gamma }^{n}}^{-}\leq PFPCAG_{\alpha ,\beta ,\gamma }^{n}\left(p_{1},p_{2},\ldots ,p_{m}\right)\leq d_{G_{\alpha ,\beta ,\gamma }^{n}}^{+},$$

*where* $d_{\Delta }^{+}=\left(\underset{i}{max}\left(\mu_{\Delta }\right),\underset{i}{min}\left(v_{\Delta }\right)\right)$ *and* $d_{\Delta }^{-}=\left(\underset{i}{min}\left(\mu_{\Delta }\right),\underset{i}{max}\left(v_{\Delta }\right)\right)$ *and* Δ *denotes* $D_{\alpha ,\beta }^{n},F_{\alpha ,\beta ,\gamma }^{n},$ $G_{\alpha ,\beta ,\gamma }^{n}.$

**Proof.**  We prove the Equation (62) holds for all *m*, and the others can be proved analogously.

From Theorem 6, we can get

$$PFPCAF_{\alpha ,\beta ,\gamma }^{n}\left(p_{1},p_{2},\ldots ,p_{m}\right)=\left(\left(1-\prod_{i=1}^{m}{\left(1-\mu_{F_{\alpha_{i},\beta_{i},\gamma_{i}}^{n}\left(p_{\sigma \left(i\right)}\right)}\right)}^{{\tilde{\omega }}_{i}}\right),\prod_{i=1}^{m}\eta_{F_{\alpha_{i},\beta_{i},\gamma_{i}}^{n}\left(p_{\sigma \left(i\right)}\right)}^{{\tilde{\omega }}_{i}},\prod_{i=1}^{m}v_{F_{\alpha_{i},\beta_{i},\gamma_{i}}^{n}\left(p_{\sigma \left(i\right)}\right)}^{{\tilde{\omega }}_{i}}\right).$$

By the definition of $d_{F_{\xi ,\zeta }^{n}}^{+},d_{F_{\xi ,\zeta }^{n}}^{-}$ we can get

$$1-\prod_{i=1}^{m}{\left(1-min\left(\mu_{F_{\alpha_{i},\beta_{i},\gamma_{i}}^{n}\left(p_{\sigma \left(i\right)}\right)}\right)\right)}^{{\tilde{\omega }}_{i}}\leq 1-\prod_{i=1}^{m}{\left(1-\mu_{F_{\alpha_{i},\beta_{i},\gamma_{i}}^{n}\left(p_{\sigma \left(i\right)}\right)}\right)}^{{\tilde{\omega }}_{i}}\leq 1-\prod_{i=1}^{m}{\left(1-max\left(\mu_{F_{\alpha_{i},\beta_{i},\gamma_{i}}^{n}\left(p_{\sigma \left(i\right)}\right)}\right)\right)}^{{\tilde{\omega }}_{i}}\;FPCAF_{\alpha ,\beta ,\gamma }^{n}\left(p_{1}^{-},p_{2}^{-},\ldots ,p_{m}^{-}\right)\leq PFPCAF_{\alpha ,\beta ,\gamma }^{n}\left(p_{1},p_{2},\ldots ,p_{m}\right)\leq FPCAF_{\alpha ,\beta ,\gamma }^{n}\left(p_{1}^{+},p_{2}^{+},\ldots ,p_{m}^{+}\right).$$

By Definition 7, we get $d_{F_{\alpha ,\beta ,\gamma }^{n}}^{-}\leq PFPCAF_{\alpha ,\beta ,\gamma }^{n}\left(p_{1},p_{2},\ldots ,p_{m}\right)\leq d_{F_{\alpha ,\beta ,\gamma }^{n}}^{+}$. ☐

By giving different values of the parameters, we get the following special cases.

**Theorem** **12.** *Let* $p_{i}=\left(\mu_{i},\eta_{i},v_{i}\right)\left(i=1,2,\ldots ,m\right)$ *be a collection of PFNs, then*

*(1)*  *If* $\omega_{i}={\tilde{\omega }}_{i}=\rho \left(A_{\sigma \left(i\right)}\right)-\rho \left(A_{\sigma \left(i-1\right)}\right),$ *then the series of PFPCA operators are all reduced to the series of picture fuzzy point averaging (PFPWA) operators. In particular, if* $m_{i}=\frac{1}{m},\left(i=1,2,\ldots ,m\right),$ *then PFPCA operators is reduced to a picture fuzzy averaging (PFA) operator, which is defined as:*
  $$PFA=\left(\sqrt{1-{\left(\prod_{i=1}^{m}\left(1-\mu_{i}^{2}\right)\right)}^{1/m}},{\left(\prod_{i=1}^{m}\eta_{i}\right)}^{1/m},{\left(\prod_{i=1}^{m}v_{i}\right)}^{1/m}\right).\;$$
*(2)*  *If* $n=0,{\tilde{\omega }}_{i}=\rho \left(A_{\sigma \left(i\right)}\right)-\rho \left(A_{\sigma \left(i-1\right)}\right),$ *and* $\rho \left(A\right)=\sum_{i=1}^{\left|A\right|}{\tilde{\omega }}_{i}$ *for all* A⊆X, *where* |A| *is the number of the elements in set A,* $\tilde{\omega }={\left({\tilde{\omega }}_{1},{\tilde{\omega }}_{2},\cdots {\tilde{\omega }}_{m}\right)}^{T},$ ${\tilde{\omega }}_{i}\in \left[0,1\right],\sum_{i=1}^{m}{\tilde{\omega }}_{i}=1$ *, then the PFPCA operator is reduced to a picture fuzzy order-weighted averaging (PFOWA) operator defined by Garg [13].*
*(3)*  *If* $n=0,{\tilde{\omega }}_{i}=\rho \left(A_{\sigma \left(i\right)}\right)-\rho \left(A_{\sigma \left(i-1\right)}\right),$ *then the series of PFPCA operators are all reduced to the series of picture fuzzy weighted averaging (PFWA) operators defined by Garg [13].*

### 4.2. Picture Fuzzy Point–Choquet Geometric Operator

**Definition** **11.** *Let Ω be the set of all PFNs, and* $p_{i}=\left(\mu_{i},\eta_{i},v_{i}\right)\left(i=1,2,\ldots ,m\right)$ *be a collection of PFNs, taking* $\alpha_{i},\beta_{i},\gamma_{i}\in \left[0,1\right]$ *. Then we define the series of PFPCG operators:* $\Omega^{m}\to \Omega$ *, if*

(64) $$F\left(C_{4}\right)\int pd\rho =PFPCGD_{\alpha ,\beta }^{n}\left(p_{1},p_{2}\cdots p_{m}\right)=\prod_{i=1}^{m}\left(\rho \left(A_{\sigma \left(i\right)}\right)-\rho \left(A_{\sigma \left(i-1\right)}\right)\right)D_{\alpha_{\sigma \left(i\right),}\beta_{\sigma \left(i\right)}}^{n}\left(p_{\sigma \left(i\right)}\right),$$

(65) $$F\left(C_{5}\right)\int pd\rho =PFPCGF_{\alpha_{,}\beta ,\gamma }^{n}\left(p_{1},p_{2}\cdots p_{m}\right)=\prod_{i=1}^{m}\left(\rho \left(A_{\sigma \left(i\right)}\right)-\rho \left(A_{\sigma \left(i-1\right)}\right)\right)F_{\alpha_{\sigma \left(i\right),}\beta_{\sigma \left(i\right)},\gamma_{\sigma \left(i\right)}}^{n}\left(p_{\sigma \left(i\right)}\right),$$

(66) $$F\left(C_{6}\right)\int pd\rho =PFPCGG_{\alpha_{,}\beta ,\gamma }^{n}\left(p_{1},p_{2}\cdots p_{m}\right)=\prod_{i=1}^{m}\left(\rho \left(A_{\sigma \left(i\right)}\right)-\rho \left(A_{\sigma \left(i-1\right)}\right)\right)G_{\alpha_{\sigma \left(i\right),}\beta_{\sigma \left(i\right)},\gamma_{\sigma \left(i\right)}}^{n}\left(p_{\sigma \left(i\right)}\right),$$

*where* σ(i) *denotes a permutation of* (1,2⋯m) *such that* $p_{\sigma \left(1\right)}\geq p_{\sigma \left(2\right)}\geq \cdots \geq p_{\sigma \left(m\right)},$ *and* $G_{\sigma \left(i\right)}$ *is the attribute corresponding to* $p_{\sigma \left(i\right)},$ $A_{\sigma \left(i\right)}=\{G_{\sigma \left(1\right)},\cdots G_{\sigma \left(i\right)}\},A_{\sigma \left(0\right)}=\phi .$

By operational laws defined in Section 2.1, we can obtain the following theorem.

**Theorem** **13.** *Let* $p_{i}=\left(\mu_{i},\eta_{i},v_{i}\right)\left(i=1,2,\ldots ,m\right)$ *be a collection of PFNs, and* σ(i) *be a permutation of* (1,2⋯m) *such that* $p_{\sigma \left(1\right)}\geq p_{\sigma \left(2\right)}\geq \cdots \geq p_{\sigma \left(m\right)},$ $G_{\sigma \left(i\right)}$ *is the attribute corresponding to* $p_{\sigma \left(i\right)},$ $A_{\sigma \left(i\right)}=\{G_{\sigma \left(1\right)},\cdots G_{\sigma \left(i\right)}\},A_{\sigma \left(0\right)}=\phi .$ *Taking* ${\tilde{\omega }}_{i}=\rho \left(A_{\sigma \left(i\right)}\right)-\rho \left(A_{\sigma \left(i-1\right)}\right),$ $\alpha_{i},\beta_{i},\gamma_{i}\in \left[0,1\right]$ *,* $\alpha_{i}+\beta_{i}+\gamma_{i}\leq 1,$ *then the aggregated values by the series of PFPCG operators are also PFNs, and*

(67) $$PFPCGD_{\alpha ,\beta }^{n}\left(p_{1},p_{2}\cdots p_{m}\right)=\;\{\prod_{i=1}^{m}{\left(\mu_{p_{\sigma \left(i\right)}}+\left(1-\gamma_{i}-\beta_{i}\right)\pi_{p_{\sigma \left(i\right)}}\right)}^{{\tilde{\omega }}_{i}},1-\prod_{i=1}^{m}{\left(1-\left(\eta_{p_{\sigma \left(i\right)}}+\beta_{i}\pi_{p_{\sigma \left(i\right)}}\right)\right)}^{{\tilde{\omega }}_{i}},\prod_{i=1}^{m}{\left(1-\left(v_{p_{\sigma \left(i\right)}}+\gamma_{i}\pi_{p_{\sigma \left(i\right)}}\right)\right)}^{{\tilde{\omega }}_{i}}\}$$

(68) $$PFPCGF_{\alpha ,\beta ,\gamma }^{n}\left(p_{1},p_{2},\ldots ,p_{m}\right)=\left(\prod_{i=1}^{m}\mu_{F_{\alpha_{i},\beta_{i},\gamma_{i}}^{n}\left(p_{\sigma \left(i\right)}\right)}^{{\tilde{\omega }}_{i}},1-\prod_{i=1}^{m}{\left(1-\eta_{F_{\alpha_{i},\beta_{i},\gamma_{i}}^{n}\left(p_{\sigma \left(i\right)}\right)}\right)}^{{\tilde{\omega }}_{i}},1-\prod_{i=1}^{m}{\left(1-v_{F_{\alpha_{i},\beta_{i},\gamma_{i}}^{n}\left(p_{\sigma \left(i\right)}\right)}\right)}^{{\tilde{\omega }}_{i}}\right)\;$$

*where*

$$\mu_{F_{\alpha_{i},\beta_{i},\gamma_{i}}^{n}\left(p_{i}\right)}=\mu_{p_{\sigma \left(i\right)}}+\alpha_{i}\pi_{p_{\sigma \left(i\right)}}\frac{1-{\left(1-\alpha_{i}-\beta_{i}-\gamma_{i}\right)}^{n}}{\alpha_{i}+\beta_{i}+\gamma_{i}},$$

$$\eta_{F_{\alpha_{i},\beta_{i},\gamma_{i}}^{n}\left(p_{i}\right)}=\eta_{p_{\sigma \left(i\right)}}+\beta_{i}\pi_{p_{\sigma \left(i\right)}}\frac{1-{\left(1-\alpha_{i}-\beta_{i}-\gamma_{i}\right)}^{n}}{\alpha_{i}+\beta_{i}+\gamma_{i}},$$

$$v_{F_{\alpha_{i},\beta_{i},\gamma_{i}}^{n}\left(p_{\sigma \left(i\right)}\right)}=v_{p_{\sigma \left(i\right)}}+\gamma_{i}\pi_{p_{\sigma \left(i\right)}}\frac{1-{\left(1-\alpha_{i}-\beta_{i}-\gamma_{i}\right)}^{n}}{\alpha_{i}+\beta_{i}+\gamma_{i}}$$

(69) $$PFPCGG_{\alpha ,\beta ,\gamma }^{n}\left(p_{1},p_{2},\ldots ,p_{m}\right)=\left(\left(1-\prod_{i=1}^{m}{\left(1-\mu_{p_{\sigma \left(i\right)}}\alpha_{i}^{n}\right)}^{{\tilde{\omega }}_{i}}\right),\prod_{i=1}^{m}{\left(\eta_{p_{\sigma \left(i\right)}}\beta_{i}^{n}\right)}^{{\tilde{\omega }}_{i}},\prod_{i=1}^{m}{\left(v_{p_{\sigma \left(i\right)}}\gamma_{i}^{n}\right)}^{{\tilde{\omega }}_{i}}\right)$$

**Theorem** **14.** *Let* $p_{i}=\left(\mu_{i},\eta_{i},v_{i}\right)\left(i=1,2,\ldots ,m\right)$ *be a collection of PFNs. Taking* k≻0 *, then*

(70) $$PFPCGD_{\alpha ,\beta }^{n}\left(p_{1}^{k},p_{2}^{k},\ldots ,p_{m}^{k}\right)={\left(PFPCGD_{\alpha ,\beta }^{n}\left(p_{1},p_{2},\ldots ,p_{m}\right)\right)}^{k},$$

(71) $$PFPCGF_{\alpha ,\beta ,\gamma }^{n}\left(p_{1}^{k},p_{2}^{k},\ldots ,p_{m}^{k}\right)={\left(PFPCGF_{\alpha ,\beta ,\gamma }^{n}\left(p_{1},p_{2},\ldots ,p_{m}\right)\right)}^{k},$$

(72) $$PFPCGG_{\alpha ,\beta ,\gamma }^{n}\left(p_{1}^{k},p_{2}^{k},\ldots ,p_{m}^{k}\right)={\left(PFPCGG_{\alpha ,\beta ,\gamma }^{n}\left(p_{1},p_{2},\ldots ,p_{m}\right)\right)}^{k}.$$

**Theorem** **15.** *Let* $p_{i}=\left(\mu_{p_{i}},\eta_{p_{i}},v_{p_{i}}\right)$ *and* $q_{i}=\left(\mu_{q_{i}},\eta_{q_{i}},v_{q_{i}}\right)\left(i=1,2,\ldots ,m\right)$ *be two collections of PFNs, then*

(73) $$PFPCGD_{\alpha ,\beta }^{n}\left(p_{1}⊗q_{1},p_{2}⊗q_{2},\ldots ,p_{m}⊗q_{m}\right)\;=PFPCGD_{\alpha ,\beta }^{n}\left(p_{1},p_{2},\ldots ,p_{m}\right)⊗PFPCGD_{\alpha ,\beta }^{n}\left(q_{1},q_{2},\ldots ,q_{m}\right),$$

(74) $$PFPCGF_{\alpha ,\beta ,\gamma }^{n}\left(p_{1}⊗q_{1},p_{2}⊗q_{2},\ldots ,p_{m}⊗q_{m}\right)\;=PFPCGF_{\alpha ,\beta ,\gamma }^{n}\left(p_{1},p_{2},\ldots ,p_{m}\right)⊗PFPCGF_{\alpha ,\beta ,\gamma }^{n}\left(q_{1},q_{2},\ldots ,q_{m}\right),$$

(75) $$PFPCGG_{\alpha ,\beta ,\gamma }^{n}\left(p_{1}⊗q_{1},p_{2}⊗q_{2},\ldots ,p_{m}⊗q_{m}\right)\;=PFPCGG_{\alpha ,\beta ,\gamma }^{n}\left(p_{1},p_{2},\ldots ,p_{m}\right)⊗PFPCGG_{\alpha ,\beta ,\gamma }^{n}\left(q_{1},q_{2},\ldots ,q_{m}\right).$$

Parallel to Theorems 9–11, the series of PFPCG operators have properties similar to PFPCA operators such as idempotency, monotonicity, and boundedness under some conditions, which are omitted in order to save space.

$$\omega_{i}={\tilde{\omega }}_{i}=\rho \left(A_{\sigma \left(i\right)}\right)-\rho \left(A_{\sigma \left(i-1\right)}\right).\;$$

### 4.3. Generalized Picture Fuzzy Point–Choquet Averaging Operator

**Definition** **12.** *Let*$p_{i}=\left(\mu_{i},\eta_{i},v_{i}\right)\left(i=1,2,\ldots ,m\right)$*be a collection of PFNs, taking*$\alpha_{i},\beta_{i},\gamma_{i}\in \left[0,1\right],\lambda ≻0$*, and*$\alpha_{i}+\beta_{i}+\gamma_{i}\leq 1$*. Then we define a series of GPFPCA operators:* Ω^m^ → Ω*, if*

(76) $$F\left(C_{7}\right)\int pd\rho =GPFPCAD_{\alpha ,\beta }^{n}\left(p_{1},p_{2}\cdots p_{n}\right)={\left(\sum_{i=1}^{m}\left(\rho \left(A_{\sigma \left(i\right)}\right)-\rho \left(A_{\sigma \left(i-1\right)}\right)\right){\left(D_{\alpha_{\sigma \left(i\right),}\beta_{\sigma \left(i\right)}}^{n}\left(p_{\sigma \left(i\right)}\right)\right)}^{\lambda }\right)}^{1/\lambda },$$

(77) $$F\left(C_{8}\right)\int pd\rho =GPFPCAF_{\alpha_{,}\beta ,\gamma }^{n}\left(p_{1},p_{2}\cdots p_{n}\right)={\left(\sum_{i=1}^{m}\left(\rho \left(A_{\sigma \left(i\right)}\right)-\rho \left(A_{\sigma \left(i-1\right)}\right)\right){\left(F_{\alpha_{\sigma \left(i\right),}\beta_{\sigma \left(i\right)},\gamma_{\sigma \left(i\right)}}^{n}\left(p_{\sigma \left(i\right)}\right)\right)}^{\lambda }\right)}^{1/\lambda },$$

(78) $$F\left(C_{9}\right)\int pd\rho =GPFPCAG_{\alpha_{,}\beta ,\gamma }^{n}\left(p_{1},p_{2}\cdots p_{n}\right)={\left(\sum_{i=1}^{m}\left(\rho \left(A_{\sigma \left(i\right)}\right)-\rho \left(A_{\sigma \left(i-1\right)}\right)\right){\left(G_{\alpha_{\sigma \left(i\right),}\beta_{\sigma \left(i\right)},\gamma_{\sigma \left(i\right)}}^{n}\left(p_{\sigma \left(i\right)}\right)\right)}^{\lambda }\right)}^{1/\lambda }.$$

*where* $G_{\sigma \left(i\right)}$ *is the attribute corresponding to* $p_{\sigma \left(i\right)},$ $A_{\sigma \left(i\right)}=\{G_{\sigma \left(1\right)},\cdots G_{\sigma \left(i\right)}\},A_{\sigma \left(0\right)}=\phi ,$ *and* σ(i) *denotes a permutation of* (1,2,…,m) *such that* $p_{\sigma \left(1\right)}\geq p_{\sigma \left(2\right)}\geq \cdots \geq p_{\sigma \left(m\right)}.$

By operational laws defined in Section 2.1, we can obtain the following theorem.

**Theorem** **16.** *Let* $p_{i}=\left(\mu_{i},\eta_{i},v_{i}\right)\left(i=1,2,\ldots ,m\right)$ *be a collection of PFNs, and* σ(i) *be a permutation of* (1,2⋯m) *such that* $p_{\sigma \left(1\right)}\geq p_{\sigma \left(2\right)}\geq \cdots \geq p_{\sigma \left(m\right)},G_{\sigma \left(i\right)}$ *is the attribute corresponding to* $p_{\sigma \left(i\right)},$ $A_{\sigma \left(0\right)}=\phi ,$ $A_{\sigma \left(i\right)}=\{G_{\sigma \left(1\right)},\cdots G_{\sigma \left(i\right)}\},$ *and taking* ${\tilde{\omega }}_{i}=\rho \left(A_{\sigma \left(i\right)}\right)-\rho \left(A_{\sigma \left(i-1\right)}\right),$ $\alpha_{i},\beta_{i},\gamma_{i}\in \left[0,1\right],$ $\alpha_{i}+\beta_{i}+\gamma_{i}\leq 1$ *, then the aggregated values by the series of GPFPCA operators are also PFNs.*

(1)

$$\begin{array}{l} GPFPCAD_{\alpha ,\beta }^{n}\left(p_{1},p_{2}\cdots p_{n}\right)=\{1-\prod_{i=1}^{m}{\left(1-{\left(\mu_{p_{\sigma \left(i\right)}}+\alpha_{i}\pi_{p_{\sigma \left(i\right)}}\right)}^{\lambda }\right)}^{{\tilde{\omega }}_{i}},\\ 1-{\left(1-\prod_{i=1}^{m}{\left(1-{\left(1-\eta_{p_{\sigma \left(i\right)}}-\left(1-\beta_{i}\right)\pi_{p_{\sigma \left(i\right)}}\right)}^{\lambda }\right)}^{{\tilde{\omega }}_{j}}\right)}^{1/\lambda },\;1-{\left(1-\prod_{i=1}^{m}{\left(1-{\left(1-v_{p_{\sigma \left(i\right)}}-\left(1-\gamma_{i}\right)\pi_{p_{\sigma \left(i\right)}}\right)}^{\lambda }\right)}^{{\tilde{\omega }}_{j}}\right)}^{1/\lambda }\} \end{array};$$

(2)

$$\begin{array}{l} GPFPCAF_{\alpha ,\beta ,\gamma }^{n}\left(p_{1},p_{2},\ldots ,p_{m}\right)=\\ \left({\left(1-\prod_{i=1}^{m}{\left(1-\mu_{F_{\alpha_{i},\beta_{i},\gamma_{i}}^{n}\left(p_{\sigma_{i}}\right)}^{\lambda }\right)}^{{\tilde{\omega }}_{i}}\right)}^{\frac{1}{\lambda }},1-{\left[1-\prod_{i=1}^{m}{\left(1-{\left(1-\eta_{F_{\alpha_{i},\beta_{i},\gamma_{i}}^{n}\left(p_{\sigma_{i}}\right)}\right)}^{\lambda }\right)}^{{\tilde{\omega }}_{i}}\right]}^{\frac{1}{\lambda }},1-{\left[1-\prod_{i=1}^{m}{\left(1-{\left(1-v_{F_{\alpha_{i},\beta_{i},\gamma_{i}}^{n}\left(p_{\sigma_{i}}\right)}\right)}^{\lambda }\right)}^{{\tilde{\omega }}_{i}}\right]}^{\frac{1}{\lambda }}\right) \end{array}$$

*where*

$$\mu_{F_{\alpha_{i},\beta_{i},\gamma_{i}}^{n}\left(p_{i}\right)}=\mu_{p_{\sigma \left(i\right)}}+\alpha_{i}\pi_{p_{\sigma \left(i\right)}}\frac{1-{\left(1-\alpha_{i}-\beta_{i}-\gamma_{i}\right)}^{n}}{\alpha_{i}+\beta_{i}+\gamma_{i}},$$

$$\eta_{F_{\alpha_{i},\beta_{i},\gamma_{i}}^{n}\left(p_{i}\right)}=\eta_{p_{\sigma \left(i\right)}}+\beta_{i}\pi_{p_{\sigma \left(i\right)}}\frac{1-{\left(1-\alpha_{i}-\beta_{i}-\gamma_{i}\right)}^{n}}{\alpha_{i}+\beta_{i}+\gamma_{i}},$$

$$v_{F_{\alpha_{i},\beta_{i},\gamma_{i}}^{n}\left(p_{\sigma \left(i\right)}\right)}=v_{p_{\sigma \left(i\right)}}+\gamma_{i}\pi_{p_{\sigma \left(i\right)}}\frac{1-{\left(1-\alpha_{i}-\beta_{i}-\gamma_{i}\right)}^{n}}{\alpha_{i}+\beta_{i}+\gamma_{i}};$$

(3) $GPFPCAF_{\alpha ,\beta ,\gamma }^{n}\left(p_{1},p_{2},\ldots ,p_{m}\right)=$

$$\left({\left(1-\prod_{i=1}^{m}{\left(1-\mu_{p_{\sigma \left(i\right)}}^{\lambda }\alpha_{i}^{n\lambda }\right)}^{{\tilde{\omega }}_{i}}\right)}^{\frac{1}{\lambda }},1-{\left[1-\prod_{i=1}^{m}{\left(1-{\left(1-\eta_{p_{\sigma \left(i\right)}}\beta_{i}^{n}\right)}^{\lambda }\right)}^{{\tilde{\omega }}_{i}}\right]}^{\frac{1}{\lambda }},1-{\left[1-\prod_{i=1}^{m}{\left(1-{\left(1-v_{p_{\sigma \left(i\right)}}\gamma_{i}^{n}\right)}^{\lambda }\right)}^{{\tilde{\omega }}_{i}}\right]}^{\frac{1}{\lambda }}\right).$$

Parallel to Theorems 9–11, the series of GPFPCA operators have properties similar to PFPCA operators such as idempotency, monotonicity, and boundedness under some conditions, which are omitted in order to save space.

### 4.4. Generalized Picture Fuzzy Point–Choquet Geometric Ooperator

**Definition** **13.** *Let*$p_{i}=\left(\mu_{i},\eta_{i},v_{i}\right)\left(i=1,2,\ldots ,m\right)$*be a collection of PFNs, taking*$\alpha_{i},\beta_{i},\gamma_{i}\in \left[0,1\right],\lambda ≻0$*, and*$\alpha_{i}+\beta_{i}+\gamma_{i}\leq 1$*. Then we define a series of GPFPCG operators:* Ω^m^ → Ω*, if*

(79) $$F\left(C_{10}\right)\int pd\rho =GPFPCGD_{\alpha ,\beta }^{n}\left(p_{1},p_{2}\cdots p_{m}\right)=\frac{1}{\lambda }\prod_{i=1}^{m}{\left(\lambda D_{\alpha_{\sigma \left(i\right),}\beta_{\sigma \left(i\right)}}^{n}\left(p_{\sigma \left(i\right)}\right)\right)}^{\left(\rho \left(A_{\sigma \left(i\right)}\right)-\rho \left(A_{\sigma \left(i-1\right)}\right)\right)},$$

(80) $$F\left(C_{11}\right)\int pd\rho =GPFPCGF_{\alpha_{,}\beta ,\gamma }^{n}\left(p_{1},p_{2}\cdots p_{m}\right)=\frac{1}{\lambda }\prod_{i=1}^{m}{\left(\lambda F_{\alpha_{\sigma \left(i\right),}\beta_{\sigma \left(i\right)}\gamma_{\sigma \left(i\right)}}^{n}\left(p_{\sigma \left(i\right)}\right)\right)}^{\left(\rho \left(A_{\sigma \left(i\right)}\right)-\rho \left(A_{\sigma \left(i-1\right)}\right)\right)},$$

(81) $$F\left(C_{12}\right)\int pd\rho =GPFPCGG_{\alpha_{,}\beta ,\gamma }^{n}\left(p_{1},p_{2}\cdots p_{m}\right)=\frac{1}{\lambda }\prod_{i=1}^{m}{\left(\lambda G_{\alpha_{\sigma \left(i\right),}\beta_{\sigma \left(i\right)}\gamma_{\sigma \left(i\right)}}^{n}\left(p_{\sigma \left(i\right)}\right)\right)}^{\left(\rho \left(A_{\sigma \left(i\right)}\right)-\rho \left(A_{\sigma \left(i-1\right)}\right)\right)}.$$

Similarly, we can obtain the following theorem:

**Theorem** **17.** *Let* $p_{i}=\left(\mu_{i},\eta_{i},v_{i}\right)\left(i=1,2,\ldots ,m\right)$ *be a collection of PFNs, and* σ(i) *be a permutation of* (1,2⋯m) *such that* $p_{\sigma \left(1\right)}\geq p_{\sigma \left(2\right)}\geq \cdots \geq p_{\sigma \left(m\right)},$ $G_{\sigma \left(i\right)}$ *is the attribute corresponding to* $p_{\sigma \left(i\right)},$ $A_{\sigma \left(0\right)}=\phi ,$ *and* $A_{\sigma \left(i\right)}=\{G_{\sigma \left(1\right)},\cdots G_{\sigma \left(i\right)}\}.$ *Taking* ${\tilde{\omega }}_{i}=\rho \left(A_{\sigma \left(i\right)}\right)-\rho \left(A_{\sigma \left(i-1\right)}\right),$ $\alpha_{i},\beta_{i},\gamma_{i}\in \left[0,1\right],$ $\alpha_{i}+\beta_{i}+\gamma_{i}\leq 1$ *, then the aggregated values by the series of GPFPCG operators are also PFNs, and*

(1)

$$\begin{array}{l} GPFPCGD_{\alpha ,\beta }^{n}\left(p_{1},p_{2}\cdots p_{n}\right)=\\ \{1-{\left(1-\prod_{i=1}^{m}{\left(1-{\left(1-\mu_{p_{\sigma \left(i\right)}}-\left(1-\alpha_{i}\right)\pi_{p_{\sigma \left(i\right)}}\right)}^{\lambda }\right)}^{{\tilde{\omega }}_{i}}\right)}^{1/\lambda },1-\prod_{i=1}^{m}{\left(1-{\left(\eta_{p_{\sigma \left(i\right)}}+\beta_{i}\pi_{p_{\sigma \left(i\right)}}\right)}^{\lambda }\right)}^{{\tilde{\omega }}_{i}},\\ 1-\prod_{i=1}^{m}{\left(1-{\left(v_{p_{\sigma \left(i\right)}}+\gamma_{i}\pi_{p_{\sigma \left(i\right)}}\right)}^{\lambda }\right)}^{{\tilde{\omega }}_{i}}\} \end{array};$$

(2)

$$\begin{array}{l} GPFPCGF_{\alpha ,\beta ,\gamma }^{n}\left(p_{1},p_{2},\ldots ,p_{m}\right)=\{1-{\left(1-\prod_{i=1}^{m}{\left(1-{\left(1-\mu_{p_{\sigma \left(i\right)}}-\left(1-\alpha_{\sigma \left(i\right)}\right)\pi_{p_{\sigma \left(i\right)}}\right)}^{\lambda }\right)}^{{\tilde{\omega }}_{i}}\right)}^{1/\lambda },\\ 1-\prod_{i=1}^{m}{\left(1-{\left(\eta_{p_{\sigma \left(i\right)}}+\beta_{\sigma \left(i\right)}\pi_{p_{\sigma \left(i\right)}}\right)}^{\lambda }\right)}^{{\tilde{\omega }}_{i}},1-\prod_{i=1}^{m}{\left(1-{\left(v_{p_{\sigma \left(i\right)}}+\gamma_{\sigma \left(i\right)}\pi_{p_{\sigma \left(i\right)}}\right)}^{\lambda }\right)}^{{\tilde{\omega }}_{i}}\} \end{array},$$

*where*

$$\mu_{F_{\alpha_{i},\beta_{i},\gamma_{i}}^{n}\left(p_{i}\right)}=\mu_{p_{\sigma \left(i\right)}}+\alpha_{i}\pi_{p_{\sigma \left(i\right)}}\frac{1-{\left(1-\alpha_{i}-\beta_{i}-\gamma_{i}\right)}^{n}}{\alpha_{i}+\beta_{i}+\gamma_{i}},$$

$$\eta_{F_{\alpha_{i},\beta_{i},\gamma_{i}}^{n}\left(p_{i}\right)}=\eta_{p_{\sigma \left(i\right)}}+\beta_{i}\pi_{p_{\sigma \left(i\right)}}\frac{1-{\left(1-\alpha_{i}-\beta_{i}-\gamma_{i}\right)}^{n}}{\alpha_{i}+\beta_{i}+\gamma_{i}},\;v_{F_{\alpha_{i},\beta_{i},\gamma_{i}}^{n}\left(p_{\sigma \left(i\right)}\right)}=v_{p_{\sigma \left(i\right)}}+\gamma_{i}\pi_{p_{\sigma \left(i\right)}}\frac{1-{\left(1-\alpha_{i}-\beta_{i}-\gamma_{i}\right)}^{n}}{\alpha_{i}+\beta_{i}+\gamma_{i}};$$

(3)

$$\begin{array}{l} GPFPCGG_{\alpha ,\beta ,\gamma }^{n}\left(p_{1},p_{2},\ldots ,p_{m}\right)=\\ \left(1-{\left[1-\prod_{i=1}^{m}{\left(1-{\left(1-\mu_{p_{{}_{\sigma \left(i\right)}}}\alpha_{{}_{i}}^{n}\right)}^{\lambda }\right)}^{\omega_{i}}\right]}^{\frac{1}{\lambda }},{\left(1-\prod_{i=1}^{m}{\left(1-\eta_{p_{{}_{\sigma \left(i\right)}}}^{\lambda }\beta_{{}_{i}}^{n\lambda }\right)}^{{\tilde{\omega }}_{i}}\right)}^{\frac{1}{\lambda }},{\left(1-\prod_{i=1}^{m}{\left(1-v_{p_{{}_{\sigma \left(i\right)}}}^{\lambda }\gamma_{{}_{i}}^{n\lambda }\right)}^{{\tilde{\omega }}_{i}}\right)}^{\frac{1}{\lambda }}\right) \end{array}.$$

Parallel to Theorems 13–15, the series of GPFPCG operators have properties such as idempotency, monotonicity, and boundedness under some conditions, which are omitted in order to save space.

In fact, the correlations of these proposed aggregation operators can be further studied. Here, we take ${\mathrm{PFPCAF}}_{\alpha ,\beta ,\gamma }^{n}$ as an example.

**Theorem** **18.** *Let* $p_{i}=\left(\mu_{i},\eta_{i},v_{i}\right)\left(i=1,2,\ldots ,m\right)$ *be a collection of PFNs, Then the operation of complement on them is as follows:*

(82) $$PFPCAF_{\alpha ,\beta ,\gamma }^{n}\left(p_{1}^{c},p_{2}^{c},\ldots ,p_{m}^{c}\right)=PFPCAF_{\alpha ,\beta ,\gamma }^{n}{\left(p_{1},p_{2},\ldots ,p_{m}\right)}^{c},$$

(83) $$PFPCGF_{\alpha ,\beta ,\gamma }^{n}\left(p_{1}^{c},p_{2}^{c},\ldots ,p_{m}^{c}\right)=PFPCGF_{\alpha ,\beta ,\gamma }^{n}{\left(p_{1},p_{2},\ldots ,p_{m}\right)}^{c},$$

(84) $$GPFPCAF_{\alpha ,\beta ,\gamma }^{n}\left(p_{1}^{c},p_{2}^{c},\ldots ,p_{m}^{c}\right)=GPFPCGF_{\alpha ,\beta ,\gamma }^{n}{\left(p_{1},p_{2},\ldots ,p_{m}\right)}^{c},$$

(85) $$GPFPCGF_{\alpha ,\beta ,\gamma }^{n}\left(p_{1}^{c},p_{2}^{c},\ldots ,p_{m}^{c}\right)=GPFPCAF_{\alpha ,\beta ,\gamma }^{n}{\left(p_{1},p_{2},\ldots ,p_{m}\right)}^{c}.$$

By Theorems 3–5, we can easily obtain the following theorems.

**Theorem** **19.** *Let* $p_{i}=\left(\mu_{i},\eta_{i},v_{i}\right)\left(i=1,2,\ldots ,m\right)$ *be a collection of PFNs, then the operation of the complement to aggregation operators is as follows:*

(86) $${\left[PFPCAF_{\alpha ,\beta ,\gamma }^{n}\left(p_{1}^{c},p_{2}^{c},\ldots ,p_{m}^{c}\right)\right]}^{c}=PFPCAF_{\alpha ,\beta ,\gamma }^{n}\left(p_{1},p_{2},\ldots ,p_{m}\right),$$

(87) $${\left[PFPCGF_{\alpha ,\beta ,\gamma }^{n}\left(p_{1}^{c},p_{2}^{c},\ldots ,p_{m}^{c}\right)\right]}^{c}=PFPCGF_{\alpha ,\beta ,\gamma }^{n}\left(p_{1},p_{2},\ldots ,p_{m}\right),$$

(88) $${\left[GPFPCGF_{\alpha ,\beta ,\gamma }^{n}\left(p_{1}^{c},p_{2}^{c},\ldots ,p_{m}^{c}\right)\right]}^{c}=GPFPCGF_{\alpha ,\beta ,\gamma }^{n}\left(p_{1},p_{2},\ldots ,p_{m}\right),$$

(89) $${\left[GPFPCAF_{\alpha ,\beta ,\gamma }^{n}\left(p_{1}^{c},p_{2}^{c},\ldots ,p_{m}^{c}\right)\right]}^{c}=GPFPCAF_{\alpha ,\beta ,\gamma }^{n}\left(p_{1},p_{2},\ldots ,p_{m}\right).$$

**Theorem** **20.** *Let* $p_{i}=\left(\mu_{i},\eta_{i},v_{i}\right)\left(i=1,2,\ldots ,m\right)$ *be a collection of PFNs, then*

(90) $$\underset{n\to \infty }{\lim}PFPCAF_{\alpha ,\beta ,\gamma }^{n}\left(p_{1},p_{2},\ldots ,p_{m}\right)=PFPCAD_{\frac{\alpha }{\alpha +\beta +\gamma },\frac{\beta }{\alpha +\beta +\gamma },}^{n}\left(p_{1},p_{2},\ldots ,p_{m}\right),$$

(91) $$\underset{n\to \infty }{\lim}PFPCGF_{\alpha ,\beta ,\gamma }^{n}\left(p_{1},p_{2},\ldots ,p_{m}\right)=PFPCGD_{\frac{\alpha }{\alpha +\beta +\gamma },\frac{\beta }{\alpha +\beta +\gamma },}^{n}\left(p_{1},p_{2},\ldots ,p_{m}\right),$$

(92) $$\underset{n\to \infty }{\lim}GPFPCAF_{\alpha ,\beta ,\gamma }^{n}\left(p_{1},p_{2},\ldots ,p_{m}\right)=GPFPCAD_{\frac{\alpha }{\alpha +\beta +\gamma },\frac{\beta }{\alpha +\beta +\gamma },}^{n}\left(p_{1},p_{2},\ldots ,p_{m}\right),$$

(93) $$\underset{n\to \infty }{\lim}GPFPCGF_{\alpha ,\beta ,\gamma }^{n}\left(p_{1},p_{2},\ldots ,p_{m}\right)=GPFPCGD_{\frac{\alpha }{\alpha +\beta +\gamma },\frac{\beta }{\alpha +\beta +\gamma },}^{n}\left(p_{1},p_{2},\ldots ,p_{m}\right).$$

**Theorem** **21.** *Let* $p_{i}=\left(\mu_{i},\eta_{i},v_{i}\right)$ *be a collection of PFNs, If* $\alpha_{i}=\frac{\mu_{p_{i}}}{\mu_{p_{i}}+\eta_{p_{i}}+v_{p_{i}}},\beta_{i}=\frac{\eta_{{}_{p_{i}}}}{\mu_{p_{i}}+\eta_{p_{i}}+v_{p_{i}}},$ $\gamma_{i}=\frac{v_{p_{i}}}{\mu_{p_{i}}+\eta_{p_{i}}+v_{p_{i}}},$ (i=1,2,…,m) *then*

(94) $$PFPCAF_{\alpha ,\beta ,\gamma }^{n}\left(p_{1},p_{2},\ldots ,p_{m}\right)=PFPCAF_{\alpha ,\beta ,\gamma }\left(p_{1},p_{2},\ldots ,p_{m}\right),$$

(95) $$PFPCGF_{\alpha ,\beta ,\gamma }^{n}\left(p_{1},p_{2},\ldots ,p_{m}\right)=PFPCGF_{\alpha ,\beta ,\gamma }\left(p_{1},p_{2},\ldots ,p_{m}\right),$$

(96) $$GPFPCAF_{\alpha ,\beta ,\gamma }^{n}\left(p_{1},p_{2},\ldots ,p_{m}\right)=GPFPCAF_{\alpha ,\beta ,\gamma }\left(p_{1},p_{2},\ldots ,p_{m}\right),$$

(97) $$GPFPCGF_{\alpha ,\beta ,\gamma }^{n}\left(p_{1},p_{2},\ldots ,p_{m}\right)=GPFPCGF_{\alpha ,\beta ,\gamma }\left(p_{1},p_{2},\ldots ,p_{m}\right).$$

In following, we discuss the differences and relationships between PFPCA, PFPCG, GPFPCA, and GPFPCG operators in detail.

In the case where λ=1, the GPFPCA operator reduces to the PFPCA operator in Definition 10, and the GPFPCG operator reduces to the PFPCG operator in Definition 11. On the other hand, the PFPCA operator is an arithmetic aggregation operator, and thus the PFPCG operator can be treated its geometric form. Similarly, GPFPCG operator is geometric form of GPFPCA operator. Since $\prod_{}^{m}x_{i}{}^{\lambda_{i}}\leq \sum_{i=1}^{m}\lambda_{i}x_{i}$ when $x_{i}≻0,\lambda_{i}≻0,\sum_{i=1}^{m}\lambda_{i}=1$, the relationships between the aggregated values obtained by the PFPCA, PFPCG, GPFPCA, and GPFPCG operators are shown as follows:

**Theorem** **22.** *Let* $p_{i}=\left(\mu_{i},\eta_{i},v_{i}\right)\left(i=1,2,\ldots ,m\right)$ *be a collection of PFNs, then*

(98) $$PFPCGF_{\alpha ,\beta ,\gamma }^{n}\left(p_{1}^{},p_{2}^{},\ldots ,p_{m}^{}\right)\leq PFPCAF_{\alpha ,\beta ,\gamma }^{n}\left(p_{1}^{},p_{2}^{},\ldots ,p_{m}^{}\right),$$

(99) $$GPFPCGF_{\alpha ,\beta ,\gamma }^{n}\left(p_{1}^{},p_{2}^{},\ldots ,p_{m}^{}\right)\leq GPFPCAF_{\alpha ,\beta ,\gamma }^{n}\left(p_{1}^{},p_{2}^{},\ldots ,p_{m}^{}\right),$$

(100) $$PFPCGF_{\alpha ,\beta ,\gamma }^{n}\left(p_{1}^{},p_{2}^{},\ldots ,p_{m}^{}\right)\leq GPFPCAF_{\alpha ,\beta ,\gamma }^{n}\left(p_{1}^{},p_{2}^{},\ldots ,p_{m}^{}\right),$$

(101) $$GPFPCGF_{\alpha ,\beta ,\gamma }^{n}\left(p_{1}^{},p_{2}^{},\ldots ,p_{m}^{}\right)\leq PFPCAF_{\alpha ,\beta ,\gamma }^{n}\left(p_{1}^{},p_{2}^{},\ldots ,p_{m}^{}\right).$$

Thus, we can conclude that the values obtained by the PFPCG operator are not bigger than the ones obtained by the PFPCA and GPFPCA. The values obtained by the GPFPCG operator are not bigger than the ones obtained by the PFPCA and GPFPCA operators for any value of $\lambda_{i}≻0$. Therefore, decision makers can select the four different operators according to their preferences and actual needs.

## 5. A New Method to Multiattribute Decision-Making with Picture Fuzzy Information

In the present section, we introduce a novel approach to MADM under the picture fuzzy environment. A typical MADM problem with picture fuzzy information can be described as: let $X=\left\{x_{1},x_{2},\ldots ,x_{m}\right\}$ be a set of alternatives, and $G=\left\{G_{1},G_{2},\ldots ,G_{s}\right\}$ be a set of attributes. Decision makers are organized to make decisions over alternatives. For attribute $G_{j}\left(j=1,2,\ldots ,s\right)$ of alternative $x_{i}\left(i=1,2,\ldots ,m\right)$, decision makers are required to use a PFN to express their preference information, which can be denoted as $p_{ij}=\left(\mu_{ij},\eta_{ij},v_{ij}\right)\left(i=1,2,\ldots ,m;j=1,2,\ldots ,s\right)$. Therefore, a picture fuzzy decision matrix can be obtained $P={\left(p_{ij}\right)}_{m\times s}$. In the following, based on the picture fuzzy aggregation operators, a novel approach to solve this problem is introduced.

**Step 1.** Generally, there are two kinds of attributes: benefit attributes and cost attributes. Therefore, the decision matrix should be normalized in the decision matrix by

(102) $$p_{ij}=\left\{ \begin{array}{l} {\left(\mu_{ij},\eta_{ij},v_{ij}\right)}_{q}\;G_{j}\in I_{1}\\ {\left(v_{ij},\eta_{ij},\mu_{ij}\right)}_{q}\;G_{j}\in I_{2} \end{array}\right.,$$

where *I*_1_ represents benefit attributes and *I*_2_ represents cost attributes. Then a normalized decision matrix can be obtained.

**Step 2.** Identify the fuzzy measure of the attributes of *G*.

**Step 3.** Rearrange the PFNs in a descending order based on the score function $S_{p}$ by Definition 9 or accuracy function $H_{p}$ by Definition 10.

**Step 4.** For alternative $x_{i}\left(i=1,2,\ldots ,m\right)$, utilize the series of PFPCA operators, or the series of PFPCG operators, or the series of GPFPCA operators, or the series of GPFPCG operators to aggregate all the attributes values. Therefore, we can get overall values $p_{i}\left(i=1,2,\ldots ,m\right)$ of alternatives.

**Step 5.** Calculate scores of $p_{i}\left(i=1,2,\ldots ,m\right)$.

**Step 6.** Rank alternatives $x_{i}\left(i=1,2,\ldots ,m\right)$ according to the rank of the corresponding overall values.

## 6. Applications in Supporting the Hierarchical Medical Treatment System with the Proposed Approach

Air pollution is currently the principal issue in the field of environmental health and PM2.5 (fine particulate matter with a aerodynamic diameter of less than 2.5 μm) has become the most important air contaminant in most cities of China, increasing health risks to the Chinese population with respect to respiratory and lung system diseases. Abundant evidence has revealed that exposure to particulate matter air pollution increases the risk of lung cancer since particulate matter with hazardous substances can enter the human body through the respiratory system and is deposited into the lung, giving rise to the damage of pulmonary function. As the research results published in The Lancet [34,35] show, among the risk factors affecting the burden of disease, ambient air pollution rank fourth as risk factor that contributed most to disability adjusted life-years. Air pollution has caused long-lasting adverse effects on respiratory health, and the adverse effect represents a substantial burden with regard to disease prevention and management.

Today, the number of patients with lung disease is soaring due to the above air pollution. In addition, China’s grassroots medical services still leave much to be desired, which to some extent force patients with lung disease to rush to large hospitals even if they only have simple lung health issues. However, the number of patients exceeds the load capacity of the large hospitals, causing great pressure. Under such circumstances, the concept of a hierarchical medical treatment system in accordance with China’s actual conditions by 2020 was introduced into the 13th Five Year Plan (2016–2020). Through the hierarchical medical treatment system, patients with different conditions can choose to go different levels of hospitals instead of all patients rushing to grade III, class A hospitals. Essentially, classifying the different degrees of diseases is a key step in pushing forward the hierarchical system. Therefore, in the present case analysis, we focus on classifying the different degrees of lung diseases to support the hierarchical medical system in China. The specific statement about the medical diagnosis problem is described as follows:

Suppose four patients, denoted by $x_{i}$ (*i* = 1, 2, 3, 4), who are possibly infected with lung diseases, need to be diagnosed and distributed according to hierarchical medical treatment system. The four patients are diagnosed from the following four symptoms (attributes) of the lung diseases: *G*_1_: vital signs, including heart rate, blood pressure, and so on; *G*_2_: body temperature (shivering and hyperthermia are two classical symptoms of pneumonia); *G*_3_: the frequency of cough; and *G*_4_: the frequency of hemoptysis. We invited a doctor who is an expert in lung diseases from a large central hospital. Then, the judgments provided by the doctor for the four patients with respect to the symptoms were represented by PFNs and the decision matrix is shown in Table 1.

With the above four diagnostic criteria for the lung diseases, the patient’s condition can be judged by the doctor. According to the degree and urgency of lung diseases, patients can be distributed to different levels and types of hospitals. Patients with severe conditions should be treated in grade III, class A hospitals, and patients with less severe symptoms should be treated in grade II hospitals. Other common illnesses can be treated in local hospitals. As mentioned in Section 2, the proposed new decision-making method does not only control the certainty of doctor’s decision data, but also deals with these situations where the decision data are correlative. Thus, the new decision-making method is suitable to be employed here.

### 6.1. Decision-Making Process

(1) The decision-making steps based on the series of PFPCA operators

**Step 1.** As all the attributes (symptoms) are benefit attributes, the decision matrix does not need to be normalized.

**Step 2.** Identify the fuzzy measure of the *n* attributes of *G*. Suppose that the fuzzy measures of attributes of *G* are given as follows:

$$\rho \left(G_{1}\right)=0.2,\rho \left(G_{2}\right)=0.3,\rho \left(G_{3}\right)=0.2,\rho \left(G_{4}\right)=0.4$$

The ρ-fuzzy measure is used to calculate the fuzzy measure of attribute sets. Firstly, according to Equation (19), the value of σ is obtained: σ=−0.237, and then the fuzzy measures of attribute sets of $G=\{G_{1},G_{2},G_{3},G_{4}\}$ can be calculated by Equation (13), shown as follows:

$$\rho \left(G_{1},G_{2}\right)=0.486,\rho \left(G_{1},G_{3}\right)=0.400,\rho \left(G_{1},G_{4}\right)=0.580\;\rho \left(G_{2},G_{4}\right)=0.680,\rho \left(G_{3},G_{4}\right)=0.581,\rho \left(G_{2},G_{3}\right)=0.486\;\rho \left(G_{1},G_{2},G_{3}\right)=0.663,\rho \left(G_{1},G_{2},G_{4}\right)=0.840,\rho \left(G_{1},G_{3},G_{4}\right)=0.754\;\rho \left(G_{2},G_{3},G_{4}\right)=0.840,\rho \left(G_{1},G_{2},G_{3},G_{4}\right)=1.$$

**Step 3.** According to Table 1, by score functions, rearrange the PFNs in descending order, shown as follows:

$$p_{1\sigma \left(1\right)}=\left(0.5,\;0.3,0.1\right),p_{1\sigma \left(2\right)}=\left(0.5,\;0.1,\;0.3\right),p_{1\sigma \left(3\right)}=\left(0.2,\;0.3,\;0.4\right),p_{1\sigma \left(4\right)}=\left(0.6,\;0.1,0.2\right),$$

$$p_{2\sigma \left(1\right)}=\left(0.6,\;0.3,\;0.1\right),p_{2\sigma \left(2\right)}=\left(0.5,\;0.2,\;0.2\right),p_{2\sigma \left(3\right)}=\left(0.4,0.4,\;0.1\right),p_{2\sigma \left(4\right)}=\left(0.7,\;0.1,\;0.2\right),$$

$$p_{3\sigma \left(1\right)}=\left(0.6,\;0.2,\;0.1\right),p_{3\sigma \left(2\right)}=\left(0.4,\;0.1,\;0.3\right),p_{3\sigma \left(3\right)}=\left(0.2,\;0.2,0.3\right),p_{3\sigma \left(4\right)}=\left(0.4,\;0.3,\;0.3\right),$$

$$p_{4\sigma \left(1\right)}=\left(0.2,\;0.3,\;0.2\right),p_{4\sigma \left(2\right)}=\left(0.6,\;0.1,\;0.3\right),p_{4\sigma \left(3\right)}=\left(0.1,\;0.3,\;0.5\right),p_{4\sigma \left(4\right)}=\left(0.1,\;0.2,0.6\right).$$

Then we can get

$$A_{1\sigma \left(1\right)}=\{G_{2}\},A_{1\sigma \left(2\right)}=\{G_{2},G_{3}\},A_{1\sigma \left(3\right)}=\{G_{2},G_{3},G_{4}\},A_{1\sigma \left(4\right)}=\{G_{1},G_{2},G_{3},G_{4}\},$$

$$A_{2\sigma \left(1\right)}=\{G_{2}\},A_{2\sigma \left(2\right)}=\{G_{2},G_{3}\},A_{2\sigma \left(3\right)}=\{G_{1},G_{2},G_{3}\},A_{2\sigma \left(4\right)}=\{G_{1},G_{2},G_{3},G_{4}\},$$

$$A_{3\sigma \left(1\right)}=\{G_{2}\},A_{2\sigma \left(2\right)}=\{G_{2},G_{3}\},A_{2\sigma \left(3\right)}=\{G_{1},G_{2},G_{3}\},A_{3\sigma \left(4\right)}=\{G_{1},G_{2},G_{3},G_{4}\},$$

$$A_{4\sigma \left(1\right)}=\{G_{4}\},A_{4\sigma \left(2\right)}=\{G_{1},G_{4}\},A_{4\sigma \left(3\right)}=\{G_{1},G_{3},G_{4}\},A_{4\sigma \left(4\right)}=\{G_{1},G_{2},G_{3},G_{4}\}.$$

Taking patientas *x*_1_ an example,

$$\rho_{1A_{\sigma \left(1\right)}}=\rho_{G_{2}}=0.3,\rho_{1A_{\sigma \left(2\right)}}-\rho_{1A_{\sigma \left(1\right)}}=\rho_{G_{2}G_{3}}-\rho_{G_{2}}=0.186,$$

$$\rho_{1A_{\sigma \left(3\right)}}-\rho_{1A_{\sigma \left(2\right)}}=\rho_{G_{2}G_{3}G_{4}}-\rho_{G_{2}G_{3}}=0.354,\rho_{1A_{\sigma \left(4\right)}}-\rho_{1A_{\sigma \left(3\right)}}=\rho_{G_{1}G_{2}G_{3}G_{4}}-\rho_{G_{2}G_{3}G_{4}}=0.16$$.

Thus, matrix of fuzzy measure is shown in Table 2:

**Step 4.** (Suppose n=1) For patients $x_{i}\left(i=1,2,3,4\right)$, utilize the series of PFPCA operators to aggregate the all the attributes values. Therefore, we can get overall values $p_{i}$ of patients.

Without loss of generality, utilizing the $PFPCAF_{\alpha ,\beta ,\gamma }^{n}$ operator in Theorem 6 to aggregate we get

$$p_{1}=\left(0.547,0.201,0.281\right),\;p_{2}=\left(0.592,0.227,0.168\right),$$

$$p_{3}=\left(0.462,0.283,0.314\right),\;p_{4}=\left(0.411,0.212,0.398\right).$$

**Step 5.** Calculating scores of $p_{i}\left(i=1,2,3,4\right)$ according to Definition 3, we can get

$$s\left(p_{1}\right)=0.266,s\left(p_{2}\right)=0.424,s\left(p_{3}\right)=0.148,s\left(p_{4}\right)=0.013.$$

Therefore, the rank of the overall values is $p_{2}≻p_{1}≻p_{3}≻p_{4}.$

**Step 6.** The rank about the patients’ conditions can be obtained according to the rank of $p_{i}$, which is $x_{2}≻x_{1}≻x_{3}≻x_{4}$. Further, the patients can be classified according to the ranking.

Therefore, the condition of patient 2 is the most serious, which means that patient 2 should be treated in a grade III, class A hospital. Meanwhile, patient 4 should be treated in a local hospital since his condition is not so serious. On the basis of the availability of the ward, patients 1 and 3 can be referred to other different types of hospitals.

(2) The decision-making steps based on the series of GPFPCA operators

**Step 1.** As all the attributes are benefit attributes, the decision matrix does not need to be normalized.

**Step 2.** (Suppose *n* = 1, *λ* = 5) For patient *x_i_* (*i* = 1,2,3,4), utilize the series of GPFPCA operators to aggregate the all the attributes values. Therefore, we can get overall values $p_{i}$ of patients.

Without loss of generality, we utilize $GPFPCAF_{\alpha ,\beta ,\gamma }^{n}$ in Theorem 16 to aggregate, and get

$$p_{1}=\left(0.575,0.443,0.265\right),p_{2}=\left(0.615,0.537,0.102\right),\;$$

$$p_{3}=\left(0.506,0.415,0.289\right),p_{4}=\left(0.506,0.265,0.366\right).$$

**Step 3.** Calculating scores of *p_i_* (*i* = 1,2,3,4) according to Definition 9, we can get

$$s\left(p_{1}\right)=0.311,s\left(p_{2}\right)=0.513,s\left(p_{3}\right)=0.217,s\left(p_{4}\right)=0.140.$$

Therefore, the rank of the overall values is $p_{2}≻p_{1}≻p_{3}≻p_{4}$.

**Step 4.** The rank about the patients’ conditions can be obtained according to the rank of $p_{i}$, which is $x_{2}≻x_{1}≻x_{3}≻x_{4}$. Further, the patients can be classified according to the ranking.

Obviously, the ranking results of the two methods proposed in this paper are same. Thus, patient 2 should be treated in a grade III, class A hospital and patient 4 should be treated in a local hospital.

### 6.2. The Influence of the Parameter Vector λ on the Final Result

The prominent characteristic of our proposed operators is not only efficiently control the certainty degree of PFS given by doctor but also model the medical diagnosis problem in a more flexible manner using an additional parameter. To reflect the influences of different values of parameter λ on the results, we utilize different parameter values of λ to rank the patients by the proposed $GPFPCAF_{\alpha ,\beta ,\gamma }^{n}$ operator, and results are shown in Table 3.

From Table 3, we can know that performance of the patients counts on the values of the parameters λ and the score values may be different for different parameters of λ in the $GPFPCAF_{\alpha ,\beta ,\gamma }^{n}$ operator. However, the ranking result for patients’ conditions is always $x_{2}≻x_{1}≻x_{3}≻x_{4}$. By further analysis, we can easily find that the score values by the $GPFPCAF_{\alpha ,\beta ,\gamma }^{n}$ operator become bigger and bigger with increasing values of λ.

**Figure 1:**
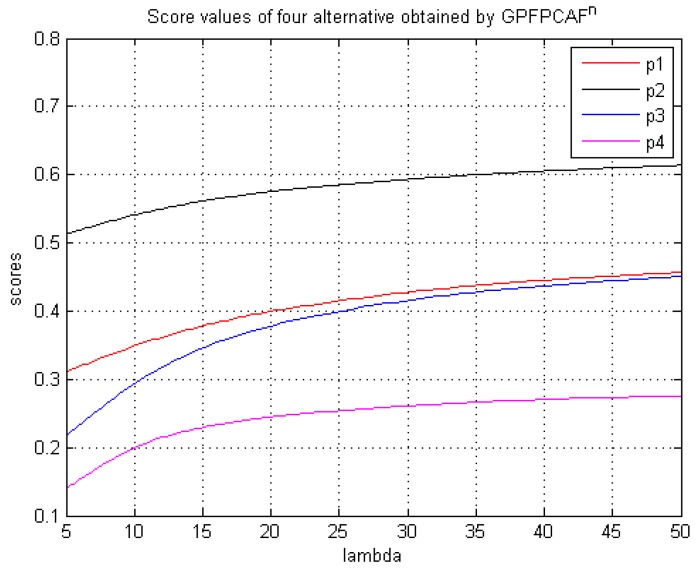
Score values when λ∈[5,50] by the ${\mathrm{GPFPCAF}}_{\alpha ,\beta ,\gamma }^{n}$ operator.

Figure 1 illustrates the scores of the patients’ conditions by the $GPFPCAF_{\alpha ,\beta ,\gamma }^{n}$ operator as assigned different values. From Figure 1, we can find that the scores of patient 3 are very close to those of patient 1 with increasing values of λ. It is easily find that that condition of patient 2 is always the most serious though the ranking results may be different for different parameters. Moreover, as the values of λ become greater and greater, the score values of the patients’ conditions are very close to the fixed values whatever the value of λ is.

### 6.3. Comparative Analysis

In order to further illustrate the effectiveness and advantages of the proposed method in this paper, we can evaluate the performance of our methods and compare it with the existing methods.

#### 6.3.1. Validity Test

We can employ the following testing criteria [36] to evaluate the performance of our methods.

**Test criterion 1:** The most serious patient will not change when one of the serious patients is replaced with another slightly serious patient without changing the relative importance of each decision criteria;

**Test criterion 2:** An effective MCDM method should follow a transitive property;

**Test criterion 3:** A combined ranking of the patients should be the same as the ranking of original problem when we decompose an MCDM problem into smaller problems.

Thus, in order to test the stability of the patients’ ranking under test criterion 1, we use a new decision matrix where diagnostic values of patients $p_{1}$, $p_{3}$, and $p_{4}$ are replaced by less serious ones denoted as ${\tilde{p}}_{1}$, ${\tilde{p}}_{3}$, and ${\tilde{p}}_{4}$ respectively, which are determined by subtracting 0.1 from the original positive degree and adding 0.1 to negative degree of patients $p_{1}$, $p_{3}$, and $p_{4}$, respectively. The new decision matrix now is shown in Table 4:

We get

$${\tilde{p}}_{1}=\left(0.448,0.201,0.386\right),{\tilde{p}}_{2}=\left(0.592,0.227,0.168\right),$$

$${\tilde{p}}_{3}=\left(0.359,0.283,0.419\right),{\tilde{p}}_{4}=\left(0.319,0.213,0.489\right)$$

and $s\left({\tilde{p}}_{1}\right)=0.062,$ $s\left({\tilde{p}}_{2}\right)=0.424,$ $s\left({\tilde{p}}_{3}\right)=-0.06,$ $s\left({\tilde{p}}_{4}\right)=-0.169$.

According to the scores of ${\tilde{p}}_{i}$, we can get $x_{2}≻{\tilde{x}}_{1}≻{\tilde{x}}_{3}≻{\tilde{x}}_{4}$. Therefore, patient 2 is in the most serious condition again, which means that our method does not change the indication of the most serious patient when non-severe patients are replaced by other more serious patients.

On the other hand, we can also add a new alternative $p_{5}$ that is in less serious condition than $p_{2}$ to test criterion 1. The alternative $p_{5}$ is obtained by subtracting 0.1 from the original positive degree and adding 0.1 to negative degree of patient $p_{2}$. Then we use the proposed method in Theorem 6 to rank $p_{i}$ (*i* = 1, 2, 3, 4, 5). We get $p_{5}=\left(0.271,0.328,0.232\right)$ and $s\left(p_{5}\right)=0.039$. According to the scores of $p_{5}$, we can get $x_{2}≻x_{1}≻x_{3}≻x_{5}≻x_{4}$, which means that the indication of patient remains unchanged when adding a new alternative.

Hence, the proposed approach is effective under test criterion 1.

In order to test the validity of our method under criterion 2 and test criterion 3, we decompose the original problem into three subproblems $\left\{p_{1},p_{2},p_{3}\right\},\left\{p_{2},p_{3},p_{4}\right\}$ and $\left\{p_{1},p_{3},p_{4}\right\}.$ Then we use same approach to solve subproblems and get the ranking corresponding to each subproblem is $x_{2}≻x_{1}≻x_{3},$ $x_{2}≻x_{3}≻x_{4}$ and $x_{1}≻x_{3}≻x_{4}$ respectively. Then a combined ranking of the patients’ condition is $x_{2}≻x_{1}≻x_{3}≻x_{4}$ which is identical to the original overall ranking of the un-decomposed problem. Therefore, the proposed method is valid under test criterion 2 and test criterion 3.

#### 6.3.2. The Advantages of the Proposed Method

To verify the effectiveness and advantages of our methods, we solve the same illustrative example by using different MADM methods including the PFWA operator, the picture fuzzy hybrid averaging (PFHA) operator, the picture fuzzy Einstein weighted average (PFEWA) operator in [13], and the picture fuzzy weighted geometric (PFWG) and picture fuzzy hybrid geometric (PFHG) operators in [12]. The ranking results are shown in Table 5.

From Table 5, we find that there are two final rankings obtained for the different eight operators; seven of them (PFHA, PFWG, PFHG, PFEWA, $PFPCAF_{\alpha ,\beta ,\gamma }^{n},PFPCGF_{\alpha ,\beta ,\gamma }^{n}$, $GPFPCAF_{\alpha ,\beta ,\gamma }^{n}$) produce the same ranking result, i.e., $x_{2}≻x_{1}≻x_{3}≻x_{4}$ and two of them (PFWA and $GPFPCGF_{\alpha ,\beta ,\gamma }^{n}$) produce a different ranking result, i.e., $x_{2}≻x_{3}≻x_{1}≻x_{4}$. Through the ranking results may be slightly different by the above MADM methods, the condition of patient 2 is always the most serious and thus should be treated in a grade III, class A hospital, and patient 4 should be treated in a local hospital. This fact verifies that the new method we proposed is effective.

In the following, we compare our proposed operators with the existing operators, such as PFWA, the picture fuzzy order weighted average (PFOWA) operator, PFHA, PFEWA, picture fuzzy Hammer weighted averaging (PFHWA) operator in [13], and PFWG, the picture fuzzy order weighted geometric (PFOWG) operator, PFHG, in [12], and the results are shown in Table 6.

From Table 6, we can find our operators have the following superiorities compared with the existing operators introduced in [12,13]:

(1) Compared with PFWA and PFWG in [12,13], the new proposed methods are more general and flexible than those provided in the existing literature.
(2) It should also be noted that the methods introduced in [12,13] are only based on the original information, and thus cannot control the certainty degree, while the new proposed methods can redistribute the membership or non-membership in PFNs according to different principles and thus can get more intensive information from the original PFS.
(3) From Table 6, it can be concluded that aggregation operators introduced in [12,13] cannot consider correlations among arguments, but the proposed aggregation operators can efficiently take the various interactions among the decision criteria into account. Furthermore, when changing the parameter *λ*, different scores are acquired shown as in Table 3, which makes decision making more flexible and can meet the needs of different types of decision makers.

Based on the comparisons and analysis above, the methods proposed in this paper can not only control the certainty of the decision data, but also deal with situations where the decision data are correlative. Thus, the proposed approaches in this paper are superior compared with other methods.

## 7. Conclusions

The hierarchical medical treatment system is an efficient way to integrate all levels of medical service system resources and release the pressure of large hospitals in China. In order to divide patients under different conditions into different levels of hospitals in the hierarchical medical treatment system, doctors need to make scientific assessments of patient’s condition based on their personal experience. In this paper, we proposed a framework to the MADM problem under the picture fuzzy environment. The analytical results show that the new approach is applicable and operational in medical diagnosis, which provides convenience for supporting the hierarchical medical treatment system in China.

There are several main contributions of this work. Firstly, it defined some point operators under picture fuzzy environment to reduce the uncertainty of doctor’s diagnosis data. By the point operators, the PFNs are translated into other PFNs, which can express more intensive information. Secondly, this paper proposed a new class of picture fuzzy point–Choquet operators including the PFPCA, PFPCG, GPFPCA, and GPFPCG operators, which can not only reduce the uncertainty of doctor’s diagnosis data, but also deal with these situations where the decision criteria (symptoms) are correlative. Thirdly, it provided a novel approach to divide patients under different conditions into different levels of hospitals based on the developed operators. Fourthly, the proposed approach has a wide range of applications and can be further applied to other MADM problems, such as pilot hospital selection, supplier selection, emergency decision making, and so on.

Although the proposed model serves better from both theoretical and practical perspectives, this research still has some deficiencies. For instance, there are four parameters in the proposed model and different parameters may lead to different results, while this paper only discusses the influences of different values of parameter λ on results. Thus, an interesting topic worthy of further study in the future is to determine the optimal parameter combination of the proposed operators. Patient condition partially depends on doctor’s judgment, which may be a little subjective when making decisions. Thus, in future study, we will consider utilizing probability theory, such as the probabilistic Pythagorean fuzzy set [37], the proportional hesitant fuzzy set [38], and the probabilistic interval-valued intuitionistic hesitant fuzzy set [39] to express doctor judgments.

## Figures and Tables

**Table 1:** The picture fuzzy decision matrix.

	*G*_1_	*G*_2_	*G*_3_	*G*_4_
*x*_1_	(0.6, 0.1, 0.2)	(0.5, 0.3, 0.1)	(0.5, 0.1, 0.3)	(0.2, 0.3, 0.4)
*x*_2_	(0.4, 0.4, 0.1)	(0.6, 0.3, 0.1)	(0.5, 0.2, 0.2)	(0.7, 0.1, 0.2)
*x*_3_	(0.2, 0.2, 0.3)	(0.6, 0.2, 0.1)	(0.4, 0.1, 0.3)	(0.4, 0.3, 0.3)
*x*_4_	(0.6, 0.1, 0.3)	(0.1, 0.2, 0.6)	(0.1, 0.3, 0.5)	(0.2, 0.3, 0.2)

**Table 2:** Matrix of fuzzy measure.

	$\rho_{A_{\sigma \left(1\right)}}$	$\rho_{A_{\sigma \left(2\right)}}-\rho_{A_{\sigma \left(1\right)}}$	$\rho_{A_{\sigma \left(3\right)}}-\rho_{A_{\sigma \left(2\right)}}$	$\rho_{A_{\sigma \left(4\right)}}-\rho_{A_{\sigma \left(3\right)}}$
*x*_1_	0.3	0.186	0.354	0.16
*x*_2_	0.3	0.186	0.177	0.337
*x*_3_	0.3	0.186	0.177	0.337
*x*_4_	0.4	0.186	0.174	0.246

**Table 3:** Rankings with different value of parameter.

Parameters	$\mathrm{Score}\;\mathrm{Value}\;\mathrm{of}\;p_{i}\;(i\;=\;1,2,3,4)$	Ranking Results
λ=5	$s\left(p_{1}\right)=0.311$ $s\left(p_{2}\right)=0.513$ $s\left(p_{3}\right)=0.217$ $s\left(p_{4}\right)=0.14$	$x_{2}≻x_{1}≻x_{3}≻x_{4}$
λ=10	$s\left(p_{1}\right)=0.350$ $s\left(p_{2}\right)=0.542$ $s\left(p_{3}\right)=0.297$ $s\left(p_{4}\right)=0.204$	$x_{2}≻x_{1}≻x_{3}≻x_{4}$
λ=15	$s\left(p_{1}\right)=0.379$ $s\left(p_{2}\right)=0.562$ $s\left(p_{3}\right)=0.348$ $s\left(p_{4}\right)=0.231$	$x_{2}≻x_{1}≻x_{3}≻x_{4}$
λ=20	$s\left(p_{1}\right)=0.401$ $s\left(p_{2}\right)=0.576$ $s\left(p_{3}\right)=0.381$ $s\left(p_{4}\right)=0.246$	$x_{2}≻x_{1}≻x_{3}≻x_{4}$
λ=30	$s\left(p_{1}\right)=0.429$ $s\left(p_{2}\right)=0.594$ $s\left(p_{3}\right)=0.418$ $s\left(p_{4}\right)=0.262$	$x_{2}≻x_{1}≻x_{3}≻x_{4}$
λ=40	$s\left(p_{1}\right)=0.446$ $s\left(p_{2}\right)=0.606$ $s\left(p_{3}\right)=0.438$ $s\left(p_{4}\right)=0.271$	$x_{2}≻x_{1}≻x_{3}≻x_{4}$
λ=50	$s\left(p_{1}\right)=0.457$ $s\left(p_{2}\right)=0.614$ $s\left(p_{3}\right)=0.451$ $s\left(p_{4}\right)=0.276$	$x_{2}≻x_{1}≻x_{3}≻x_{4}$

**Table 4:** The new picture fuzzy decision matrix.

	*G*_1_	*G*_2_	*G*_3_	*G*_4_
*x*_1_	(0.5, 0.1,0.1)	(0.4, 0.3,0.2)	(0.4, 0.1, 0.4)	(0.1, 0.3, 0.5)
*x*_2_	(0.4, 0.4, 0.1)	(0.6, 0.3, 0.1)	(0.5, 0.2, 0.2)	(0.7, 0.1, 0.2)
*x*_3_	(0.1, 0.2, 0.4)	(0.5, 0.2, 0.2)	(0.3, 0.1, 0.2)	(0.3, 0.3, 0.4)
*x*_4_	(0.5, 0.1, 0.4)	(0.1, 0.2, 0.7)	(0.1, 0.3, 0.6)	(0.1, 0.3, 0.3)

**Table 5:** Comparison of rankings with different aggregation operators.

Approaches	Score Value of *X*_i_ (*i* = 1, 2, 3, 4)	Ranking
Approach based on the PFWA operator [13]	$s\left(p_{1}\right)=0.199$ $s\left(p_{2}\right)=0.463$ $s\left(p_{3}\right)=0.222$ $s\left(p_{4}\right)=-0.061$	$x_{2}≻x_{1}≻x_{3}≻x_{4}$
Approach based on the PFHA operator [13]	$s\left(p_{1}\right)=0.239$ $s\left(p_{2}\right)=0.407$ $s\left(p_{3}\right)=0.171$ $s\left(p_{4}\right)=-0.108$	$x_{2}≻x_{1}≻x_{3}≻x_{4}$
Approach based on the PFWG operator [12]	$s\left(p_{1}\right)=0.243$ $s\left(p_{2}\right)=0.402$ $s\left(p_{3}\right)=0.103$ $s\left(p_{4}\right)=-0.061$	$x_{2}≻x_{1}≻x_{3}≻x_{4}$
Approach based on the PFHG operator [12]	$s\left(p_{1}\right)=0.327$ $s\left(p_{2}\right)=0.413$ $s\left(p_{3}\right)=0.279$ $s\left(p_{4}\right)=0.133$	$x_{2}≻x_{1}≻x_{3}≻x_{4}$
Approach based on the PFEWA operator [13]	$s\left(p_{1}\right)=0.261$ $s\left(p_{2}\right)=0.425$ $s\left(p_{3}\right)=0.139$ $s\left(p_{4}\right)=0.005$	$x_{2}≻x_{1}≻x_{3}≻x_{4}$
$\mathrm{Approach}\;\mathrm{based}\;\mathrm{on}\;PFPCAF_{\alpha ,\beta ,\gamma }^{n}$ operator (in this paper)	$s\left(p_{1}\right)=0.265$ $s\left(p_{2}\right)=0.424$ $s\left(p_{3}\right)=0.148$ $s\left(p_{4}\right)=0.013$	$x_{2}≻x_{1}≻x_{3}≻x_{4}$
$\mathrm{Approach}\;\mathrm{based}\;\mathrm{on}\;\mathrm{the}\;PFPCGF_{\alpha ,\beta ,\gamma }^{n}$ operator (in this paper)	$s\left(p_{1}\right)=0.204$ $s\left(p_{2}\right)=0.399$ $s\left(p_{3}\right)=0.095$ $s\left(p_{4}\right)=0.051$	$x_{2}≻x_{1}≻x_{3}≻x_{4}$
$\mathrm{Approach}\;\mathrm{based}\;\mathrm{on}\;\mathrm{the}\;GPFPCAF_{\alpha ,\beta ,\gamma }^{n}$ operator (in this paper)	$s\left(p_{1}\right)=0.310$ $s\left(p_{2}\right)=0.513$ $s\left(p_{3}\right)=0.217$ $s\left(p_{4}\right)=0.140$	$x_{2}≻x_{1}≻x_{3}≻x_{4}$
$\mathrm{Approach}\;\mathrm{based}\;\mathrm{on}\;\mathrm{the}\;GPFPCGF_{\alpha ,\beta ,\gamma }^{n}$ operator (in this paper)	$s\left(p_{1}\right)=0.095$ $s\left(p_{2}\right)=0.358$ $s\left(p_{3}\right)=0.111$ $s\left(p_{4}\right)=-0.091$	$x_{2}≻x_{3}≻x_{1}≻x_{4}$

**Table 6:** The comparison of different operators.

Aggregation Operators	Whether It Can Consider Correlations among Arguments	Whether It Can Control the Certainty of PFNs	Flexible (Whether There Is a Parameter to Reflect Preferences)
PFWA [13]	No	No	No
PFOWA [13]	No	No	No
PFHA [13]	No	No	No
PFWG [12]	No	No	No
PFOWG [12]	No	No	No
PFHG [12]	No	No	No
PFEWA [13]	No	No	No
PFHA [13]	No	No	No
PFPCA	Yes	Yes	Yes
PFPCG	Yes	Yes	Yes
GPFPCA	Yes	Yes	Yes
GPFPCG	Yes	Yes	Yes

## Appendix A. Proof of Theorem 2

**Proof.** We prove Equation (28) holds for all *n*, and the others can be proved analogously. From Definition 5, we get $F_{\alpha ,\beta ,\gamma }\left(p\right)$, transform a PFN into another PFN, and

$$\pi_{F_{\alpha ,\beta ,\gamma }^{n}}=1-\left(\mu_{p}+\alpha \pi_{p}\right)-\left(\eta_{p}+\beta \pi_{p}\right)-\left(v_{p}+\gamma \pi_{p}\right)=\left(1-\mu_{p}-\eta_{p}-v_{p}\right)\left(1-\alpha -\beta -\gamma \right)=\pi_{p}\left(1-\alpha -\beta -\gamma \right)$$

Since $F_{\alpha ,\beta ,\gamma }^{2}\left(p\right)=F_{\alpha ,\beta ,\gamma }\left(F_{\alpha ,\beta ,\gamma }\left(p\right)\right)$, we have

$$F_{\alpha ,\beta ,\gamma }^{2}\left(p\right)=F_{\alpha ,\beta ,\gamma }\left(F_{\alpha ,\beta ,\gamma }\left(p\right)\right)=\left\{\left(\mu_{p}+\alpha \pi_{p}+\alpha \pi_{F_{\alpha ,\beta ,\gamma }^{}}\right),\left(\eta_{p}+\beta \pi_{p}+\beta \pi_{F_{\alpha ,\beta ,\gamma }^{}}\right),\left(v_{p}+\gamma \pi_{p}+\gamma \pi_{F_{\alpha ,\beta ,\gamma }^{}}\right)\right\}.$$

Thus

$$\pi_{F_{\xi ,\zeta }^{2}\left(\gamma \right)}=\left(1-\left[\mu_{p}+\alpha \pi_{p}+\alpha \pi_{F_{\alpha ,\beta ,\gamma }^{}}\right]-\left[\eta_{p}+\beta \pi_{\gamma }^{q}+\beta \pi_{F_{\alpha ,\beta ,\gamma }^{}}\right]-\left[v_{p}+\gamma \pi_{\gamma }^{q}+\gamma \pi_{F_{\alpha ,\beta ,\gamma }^{}}\right]\right)\;=\left(\left(1-\mu_{p}-\eta_{p}-v_{p}\right)-\left(\alpha +\beta +\gamma \right)\pi_{p}-\left(\alpha +\beta +\gamma \right)\left(1-\alpha -\beta -\gamma \right)\pi_{p}\right)\;=\pi_{p}{\left(1-\alpha -\beta -\gamma \right)}^{2}$$

. Since $F_{\alpha ,\beta ,\gamma }^{3}\left(p\right)=F_{\alpha ,\beta ,\gamma }\left(F_{\alpha ,\beta ,\gamma }^{2}\left(p\right)\right)$, we have

$$F_{\alpha ,\beta ,\gamma }^{3}\left(p\right)=F_{\alpha ,\beta ,\gamma }\left(F_{\alpha ,\beta ,\gamma }^{2}\left(p\right)\right)=\{\left(\mu_{p}+\alpha \pi_{p}+\alpha \left(1-\alpha -\beta -\gamma \right)\pi_{p}+\right)+\alpha {\left(1-\alpha -\beta -\gamma \right)}^{2}\pi_{p},\;\left(\eta_{p}+\beta \pi_{p}+\beta \left(1-\alpha -\beta -\gamma \right)\pi_{p}\right)+\beta {\left(1-\alpha -\beta -\gamma \right)}^{2}\pi_{p},\;\left(v_{p}+\gamma \pi_{p}+\gamma \left(1-\alpha -\beta -\gamma \right)\pi_{p}\right)+\gamma {\left(1-\alpha -\beta -\gamma \right)}^{2}\pi_{p}\}$$

and $\pi_{F_{\alpha ,\beta ,\gamma }^{3}\left(p\right)}=\pi_{p}{\left(1-\alpha -\beta -\gamma \right)}^{3}$. Similarly, we have

$$\begin{array}{l} F_{\alpha ,\beta ,\gamma }^{n}\left(p\right)=F_{\alpha ,\beta ,\gamma }\left(F_{\alpha ,\beta ,\gamma }^{n-1}\left(p\right)\right)\\ =\{\left(\left(\mu_{p}+\alpha \pi_{p}+\left(1-\alpha -\beta -\gamma \right)\alpha \pi_{p}\right)+{\left(1-\alpha -\beta -\gamma \right)}^{2}\alpha \pi_{p}+\cdots +{\left(1-\alpha -\beta -\gamma \right)}^{n-1}\alpha \pi_{p}\right),\\ \left(\eta_{p}+\beta \pi_{p}+\left(1-\alpha -\beta -\gamma \right)\beta \pi_{p}\right)+{\left(1-\alpha -\beta -\gamma \right)}^{2}\phi \pi_{p}+\cdots +{\left(1-\alpha -\beta -\gamma \right)}^{n-1}\beta \pi_{p},\\ \left(\left(v_{p}+\gamma \pi_{p}+\left(1-\alpha -\beta -\gamma \right)\gamma \pi_{p}\right)+{\left(1-\alpha -\beta -\gamma \right)}^{2}\gamma \pi_{p}+\cdots +{\left(1-\alpha -\beta -\gamma \right)}^{n-1}\gamma \pi_{p}\right)\}\\ =\{\mu_{p}+\alpha \pi_{p}\frac{1-{\left(1-\alpha -\beta -\gamma \right)}^{n}}{\alpha +\beta +\gamma },\eta_{p}+\beta \pi_{p}\frac{1-{\left(1-\alpha -\beta -\gamma \right)}^{n}}{\alpha +\beta +\gamma },v_{p}+\gamma \pi_{p}\frac{1-{\left(1-\alpha -\beta -\gamma \right)}^{n}}{\alpha +\beta +\gamma }\}. \end{array}$$

. Therefore, Equation (28) holds, which completes the proof. ☐

## Appendix B. Proof of Theorem 6

**Proof.** We prove the Equation (47) holds for all *m*, and the others can be proved analogously. (i) We first prove $PFPCAF_{\alpha ,\beta ,\gamma }^{n}$ is also a PFN. As $0\leq \mu_{i},\eta_{i},v_{i}\leq 1,$ $0\leq \mu_{i}+\eta_{i}+v_{i}\leq 1$, and

$$\mu_{F_{\alpha_{i},\beta_{i},\gamma_{i}}^{n}\left(p_{\sigma \left(i\right)}\right)}=\mu_{p_{\sigma \left(i\right)}}+\alpha_{i}\pi_{p_{\sigma \left(i\right)}}\frac{1-{\left(1-\alpha_{i}-\beta_{i}-\gamma_{i}\right)}^{n}}{\alpha_{i}+\beta_{i}+\gamma_{i}},\;$$

$$\eta_{F_{\alpha_{i},\beta_{i},\gamma_{i}}^{n}\left(p_{i}\right)}=\eta_{p_{\sigma \left(i\right)}}+\beta_{i}\pi_{p_{\sigma \left(i\right)}}\frac{1-{\left(1-\alpha_{i}-\beta_{i}-\gamma_{i}\right)}^{n}}{\alpha_{i}+\beta_{i}+\gamma_{i}},$$

$$v_{F_{\alpha_{i},\beta_{i},\gamma_{i}}^{n}\left(p_{i}\right)}=v_{p_{\sigma \left(i\right)}}+\gamma_{i}\pi_{p_{\sigma \left(i\right)}}\frac{1-{\left(1-\alpha_{i}-\beta_{i}-\gamma_{i}\right)}^{n}}{\alpha_{i}+\beta_{i}+\gamma_{i}}.$$

Thus $0\leq {\prod_{i=1}^{m}\left(1-\mu_{F_{\alpha_{i},\beta_{i},\gamma_{i}}^{n}\left(p_{\sigma \left(i\right)}\right)}\right)}^{{\tilde{\omega }}_{i}}\leq 1$, we get

$$0\leq \left(1-{\prod_{i=1}^{m}\left(1-\mu_{F_{\alpha_{i},\beta_{i},\gamma_{i}}^{n}\left(p_{\sigma \left(i\right)}\right)}\right)}^{{\tilde{\omega }}_{i}}\right)\leq 1\;\mathrm{and}\;0\leq \prod_{i=1}^{m}v_{F_{\alpha_{i},\beta_{i},\gamma_{i}}^{n}\left(p_{\sigma \left(i\right)}\right)}^{{\tilde{\omega }}_{i}}\leq 1$$

Again

$$\begin{array}{l} 1-{\prod_{i=1}^{m}\left(1-\mu_{F_{\alpha_{i},\beta_{i},\gamma_{i}}^{n}\left(p_{i}\right)}\right)}^{{\tilde{\omega }}_{i}}+\prod_{i=1}^{m}\eta_{F_{\alpha_{i},\beta_{i},\gamma_{i}}^{n}\left(p_{i}\right)}^{{\tilde{\omega }}_{i}}+\prod_{i=1}^{m}v_{F_{\alpha_{i},\beta_{i},\gamma_{i}}^{n}\left(p_{i}\right)}^{{\tilde{\omega }}_{i}}\leq \\ 1-{\prod_{i=1}^{m}\left(1-\eta_{F_{\alpha_{i},\beta_{i},\gamma_{i}}^{n}\left(p_{\sigma \left(i\right)}\right)}^{{\tilde{\omega }}_{i}}-\nu_{F_{\alpha_{i},\beta_{i},\gamma_{i}}^{n}\left(p_{\sigma \left(i\right)}\right)}^{{\tilde{\omega }}_{i}}\right)}^{{\tilde{\omega }}_{i}}+\prod_{i=1}^{m}\eta_{F_{\alpha_{i},\beta_{i},\gamma_{i}}^{n}\left(p_{\sigma \left(i\right)}\right)}^{{\tilde{\omega }}_{i}}+\prod_{i=1}^{m}\nu_{F_{\alpha_{i},\beta_{i},\gamma_{i}}^{n}\left(p_{\sigma \left(i\right)}\right)}^{{\tilde{\omega }}_{i}}=1 \end{array}$$

. Hence, $PFPCAF_{\alpha ,\beta ,\gamma }^{n}$ is a PFN according to Definition 5. (ii) Next, we prove Equation (47) by using mathematical induction on m. When m=2, by the operational law (18) and (19) in Section 2.2, we have

$$\begin{array}{c} PFPCAF_{\alpha ,\beta ,\gamma }^{n}\left(p_{1},p_{2}\right)={\tilde{\omega }}_{1}p_{1}⊕{\tilde{\omega }}_{2}p_{2}\\ =\left(\left(1-{\left(1-\mu_{F_{\alpha_{1},\beta_{1}\gamma_{1}}^{n}\left(p_{1}\right)}\right)}^{{\tilde{\omega }}_{1}}\right),\eta_{F_{\alpha_{1},\beta_{1},\gamma_{1}}^{n}\left(p_{1}\right)}^{{\tilde{\omega }}_{1}},v_{F_{\alpha_{1},\beta_{1}\gamma_{1}}^{n}\left(p_{1}\right)}^{{\tilde{\omega }}_{1}}\right)⊕\left(\left(1-{\left(1-\mu_{F_{\alpha_{2},\beta_{2},\gamma_{2}}^{n}\left(p_{2}\right)}\right)}^{{\tilde{\omega }}_{{}_{2}}}\right),\eta_{F_{\alpha_{2},\beta_{2},\gamma_{2}}^{n}\left(p_{2}\right)}^{{\tilde{\omega }}_{1}},v_{F_{\alpha_{2},\beta_{2},\gamma_{2}}^{n}\left(p_{2}\right)}^{{\tilde{\omega }}_{2}}\right)\\ =\{\left(1-{\left(1-\mu_{F_{\alpha_{1},\beta_{1}\gamma_{1}}^{n}\left(p_{1}\right)}\right)}^{{\tilde{\omega }}_{1}}+1-{\left(1-\mu_{F_{\alpha_{2},\beta_{2},\gamma_{2}}^{n}\left(p_{2}\right)}\right)}^{{\tilde{\omega }}_{{}_{2}}}-\left(1-{\left(1-\mu_{F_{\alpha_{1},\beta_{1}\gamma_{1}}^{n}\left(p_{1}\right)}\right)}^{{\tilde{\omega }}_{1}}\right)\left(1-{\left(1-\mu_{F_{\alpha_{2},\beta_{2},\gamma_{2}}^{n}\left(p_{2}\right)}\right)}^{{\tilde{\omega }}_{{}_{2}}}\right)\right),\\ \eta_{F_{\alpha_{1},\beta_{1},\gamma_{1}}^{n}\left(p_{1}\right)}^{{\tilde{\omega }}_{1}}\eta_{F_{\alpha_{2},\beta_{2},\gamma_{2}}^{n}\left(p_{2}\right)}^{{\tilde{\omega }}_{1}},v_{F_{\alpha_{1},\beta_{1},\gamma_{1}}^{n}\left(p_{1}\right)}^{{\tilde{\omega }}_{1}}v_{F_{\alpha_{2},\beta_{2},\gamma_{2}}^{n}\left(p_{2}\right)}^{{\tilde{\omega }}_{2}}\}\\ =\left(1-{\left(1-\mu_{F_{\alpha_{1},\beta_{1}\gamma_{1}}^{n}\left(p_{1}\right)}\right)}^{{\tilde{\omega }}_{1}}{\left(1-\mu_{F_{\alpha_{2},\beta_{2},\gamma_{2}}^{n}\left(p_{2}\right)}\right)}^{{\tilde{\omega }}_{{}_{2}}},\eta_{F_{\alpha_{1},\beta_{1},\gamma_{1}}^{n}\left(p_{1}\right)}^{{\tilde{\omega }}_{1}}\eta_{F_{\alpha_{2},\beta_{2},\gamma_{2}}^{n}\left(p_{2}\right)}^{{\tilde{\omega }}_{1}},v_{F_{\alpha_{1},\beta_{1},\gamma_{1}}^{n}\left(p_{1}\right)}^{{\tilde{\omega }}_{1}}v_{F_{\alpha_{2},\beta_{2},\gamma_{2}}^{n}\left(p_{2}\right)}^{{\tilde{\omega }}_{2}}\}\right)\\ =\left(1-\prod_{i=1}^{2}{\left(1-\mu_{F_{\alpha_{i},\beta_{i},\gamma_{i}}^{n}\left(p_{\sigma \left(i\right)}\right)}\right)}^{{\tilde{\omega }}_{i}},\prod_{i=1}^{2}\eta_{F_{\alpha_{i},\beta_{i},\gamma_{i}}^{n}\left(p_{\sigma \left(i\right)}\right)}^{{\tilde{\omega }}_{i}},\prod_{i=1}^{2}\nu_{F_{\alpha_{i},\beta_{i},\gamma_{i}}^{n}\left(p_{\sigma \left(i\right)}\right)}^{{\tilde{\omega }}_{i}}\right) \end{array}.$$

Thus, result is true for m=2. If Equation (47) holds for m=k, that is

$$PFPCAF_{\alpha ,\beta ,\gamma }^{n}\left(p_{1},p_{2},\ldots ,p_{k}\right)=\left(1-\prod_{i=1}^{k}{\left(1-\mu_{F_{\alpha_{i},\beta_{i},\gamma_{i}}^{n}\left(p_{\sigma \left(i\right)}\right)}\right)}^{{\tilde{\omega }}_{i}},\prod_{i=1}^{k}\eta_{F_{\alpha_{i},\beta_{i},\gamma_{i}}^{n}\left(p_{\sigma \left(i\right)}\right)}^{{\tilde{\omega }}_{i}},\prod_{i=1}^{k}v_{F_{\alpha_{i},\beta_{i},\gamma_{i}}^{n}\left(p_{\sigma \left(i\right)}\right)}^{{\tilde{\omega }}_{i}}\right),$$

then, when m=k+1, by the operational laws (7) and (9) in Section 2.2, we have

$$\begin{array}{l} PFPCAF_{\alpha ,\beta ,\gamma }^{n}\left(p_{1},p_{2},\ldots ,p_{k+1}\right)=PFPCAF_{\alpha ,\beta ,\gamma }^{n}\left(p_{1},p_{2},\ldots ,p_{k}\right)⊕{\tilde{\omega }}_{k+1}F_{\alpha ,\beta ,\gamma }^{n}\left(p_{k+1}\right)\\ =\left(1-\prod_{i=1}^{k}{\left(1-\mu_{F_{\alpha_{i},\beta_{i},\gamma_{i}}^{n}\left(p_{\sigma \left(i\right)}\right)}\right)}^{{\tilde{\omega }}_{i}},\prod_{i=1}^{k}\eta_{F_{\alpha_{i},\beta_{i},\gamma_{i}}^{n}\left(p_{\sigma \left(i\right)}\right)}^{{\tilde{\omega }}_{i}},\prod_{i=1}^{k}v_{F_{\alpha_{i},\beta_{i},\gamma_{i}}^{n}\left(p_{\sigma \left(i\right)}\right)}^{{\tilde{\omega }}_{i}}\right)\\ \;⊕\left(1-{\left(1-\mu_{F_{\alpha_{k+1},\beta_{k+1},\gamma_{k+1}}^{n}\left(p_{\sigma \left(k+1\right)}\right)}\right)}^{{\tilde{\omega }}_{i}},\eta_{F_{\alpha_{k+1},\beta_{k+1},\gamma_{k+1}}^{n}\left(p_{\sigma \left(k+1\right)}\right)}^{{\tilde{\omega }}_{k+1}},v_{F_{\alpha_{k+1},\beta_{k+1},\gamma_{k+1}}^{n}\left(p_{\sigma \left(k+1\right)}\right)}^{{\tilde{\omega }}_{k+1}}\right)\\ =\{(1-\prod_{i=1}^{k}{\left(1-\mu_{F_{\alpha_{i},\beta_{i},\gamma_{i}}^{n}\left(p_{\sigma \left(i\right)}\right)}\right)}^{{\tilde{\omega }}_{i}}+1-{\left(1-\mu_{F_{\alpha_{k+1},\beta_{k+1},\gamma_{k+1}}^{n}\left(p_{k+1}\right)}\right)}^{{\tilde{\omega }}_{{}_{k+1}}}-\\ \left(1-\prod_{i=1}^{k}{\left(1-\mu_{F_{\alpha_{i},\beta_{i},\gamma_{i}}^{n}\left(p_{\sigma \left(i\right)}\right)}\right)}^{{\tilde{\omega }}_{i}}\right)\left(1-{\left(1-\mu_{F_{\alpha_{k+1},\beta_{k+1},\gamma_{k+1}}^{n}\left(p_{k+1}\right)}\right)}^{{\tilde{\omega }}_{{}_{k+1}}}\right)),\\ \;\prod_{i=1}^{k}\eta_{F_{\alpha_{i},\beta_{i},\gamma_{i}}^{n}\left(p_{i}\right)}^{{\tilde{\omega }}_{i}}\eta_{F_{\alpha_{k+1},\beta_{k+1},\gamma_{k+1}}^{n}\left(p_{\sigma \left(i\right)}\right)}^{{\tilde{\omega }}_{k+1}},\prod_{i=1}^{k}v_{F_{\alpha_{i},\beta_{i},\gamma_{i}}^{n}\left(p_{\sigma \left(i\right)}\right)}^{{\tilde{\omega }}_{i}}v_{F_{\alpha_{k+1},\beta_{k+1},\gamma_{k+1}}^{n}\left(p_{\sigma \left(i\right)}\right)}^{{\tilde{\omega }}_{k+1}}\}\\ =\left(\left(1-\prod_{i=1}^{k+1}{\left(1-\mu_{F_{\alpha_{i},\beta_{i},\gamma_{i}}^{n}\left(p_{\sigma \left(i\right)}\right)}\right)}^{{\tilde{\omega }}_{i}}\right),\prod_{i=1}^{k+1}\eta_{F_{\alpha_{i},\beta_{i},\gamma_{i}}^{n}\left(p_{\sigma \left(i\right)}\right)}^{{\tilde{\omega }}_{i}},\prod_{i=1}^{k+1}v_{F_{\alpha_{i},\beta_{i},\gamma_{i}}^{n}\left(p_{\sigma \left(i\right)}\right)}^{{\tilde{\omega }}_{i}}\right), \end{array}$$

Thus, Equation (47) holds for m=k+1. Therefore, Equation (47) holds for all m, which completes the proof. ☐
